# The clinical and molecular significance associated with STING signaling in breast cancer

**DOI:** 10.1038/s41523-021-00283-z

**Published:** 2021-06-25

**Authors:** Eileen E. Parkes, Matthew P. Humphries, Elaine Gilmore, Fatima A. Sidi, Victoria Bingham, Su M. Phyu, Stephanie Craig, Catherine Graham, Joseph Miller, Daryl Griffin, Manuel Salto-Tellez, Stephen F. Madden, Richard D. Kennedy, Samuel F. Bakhoum, Stephen McQuaid, Niamh E. Buckley

**Affiliations:** 1 Department of Oncology, Medical Sciences Division, University of Oxford, Oxford, UK; 2 Patrick G. Johnston Centre for Cancer Research, Queen’s University Belfast, Belfast, Northern Ireland UK; 3 Precision Medicine Centre of Excellence, Queen’s University Belfast, Belfast, Northern Ireland UK; 4 School of Pharmacy, Queen’s University Belfast, Belfast, Northern Ireland UK; 5 Department of Cellular Pathology, Belfast Health and Social Care Trust, Belfast, Northern Ireland UK; 6 Integrated Pathology Programme, Division of Molecular Pathology, The Institute of Cancer Research, London, UK; 7 Data Science Centre, RCSI University of Medicine and Health Sciences, Dublin, Ireland UK; 8 Human Oncology and Pathogenesis Program, Memorial Sloan Kettering Cancer Center, New York, NY USA; 9 Northern Ireland Biobank, Patrick G. Johnston Centre for Cancer Research, Queen’s University Belfast, Belfast, Northern Ireland UK

**Keywords:** Cancer microenvironment, Tumour immunology, Tumour biomarkers

## Abstract

STING signaling in cancer is a crucial component of response to immunotherapy and other anti-cancer treatments. Currently, there is no robust method of measuring STING activation in cancer. Here, we describe an immunohistochemistry-based assay with digital pathology assessment of STING in tumor cells. Using this novel approach in estrogen receptor-positive (ER+) and ER- breast cancer, we identify perinuclear-localized expression of STING (pnSTING) in ER+ cases as an independent predictor of good prognosis, associated with immune cell infiltration and upregulation of immune checkpoints. Tumors with low pnSTING are immunosuppressed with increased infiltration of “M2”-polarized macrophages. In ER- disease, pnSTING does not appear to have a significant prognostic role with STING uncoupled from interferon responses. Importantly, a gene signature defining low pnSTING expression is predictive of poor prognosis in independent ER+ datasets. Low pnSTING is associated with chromosomal instability, *MYC* amplification and mTOR signaling, suggesting novel therapeutic approaches for this subgroup.

## Introduction

Avoiding immune destruction and tumor-promoting inflammation are immune hallmarks of cancer, with the innate immune cyclic GMP-AMP synthase (cGAS) - STimulator of INterferon Genes (STING) pathway involved in both.^1–3^ The cGAS-STING pathway is a focal point of innate immune responses, activated when cGAS detects cytosolic DNA, producing 2′3′cGAMP and resulting in subsequent stimulation of STING and downstream TBK1-IRF3 and NFκB-RelB pathways.^4^

Activation of STING in response to DNA damaging therapies such as ionizing radiation has been reported as essential for a direct antitumor and abscopal response, as well as implicated in subsequent inflammation-mediated radioresistance.^5–7^ Activation of the STING pathway via cGAS has been identified as a prerequisite for response to immune checkpoint blockade (ICB).^8^ STING agonists, where delivery of intratumoral cyclic dinucleotides activate STING responses within the tumor microenvironment, (TME) have demonstrated synergy with ICB in preclinical models.^9,10^ Trials are ongoing to determine the safety and efficacy of STING agonists in combination with ICB in advanced metastatic cancer.^11,12^

We previously identified a STING-driven chemokine response signature in DNA repair-deficient breast cancers, which was associated with improved outcome in the context of standard-of-care chemotherapy.^13^ However, chronic stimulation of cGAS-STING via micronuclei in chromosomally unstable cancers results in inactivation of interferons and instead promotes downstream NFκB-RelB-mediated metastasis.^3^ The intrinsic STING activation or suppression inherent in cancer is a therefore a potential predictive biomarker for immune or other therapies. Furthermore, identifying immune and gene expression correlates with STING activation could suggest rational combination approaches in STING-agonist resistant cancers. Expression of *TMEM173*, encoding STING, moderately correlates with immune infiltration but only poorly correlates with expression of downstream components TBK1 and IRF3, suggesting that *TMEM173* expression may be a weak indicator of STING activity.^14^ A specific STING activation assay would be a valuable tool in addressing these important questions. However, given the diversity of downstream chemokine responses, transient nature of TBK1-IRF3 activation and crosstalk with other nucleic acid sensing pathways, such an approach has been challenging to develop.

## Results

### Perinuclear STING expression predicts prognosis in ER positive breast cancer

**Fig. 1:**
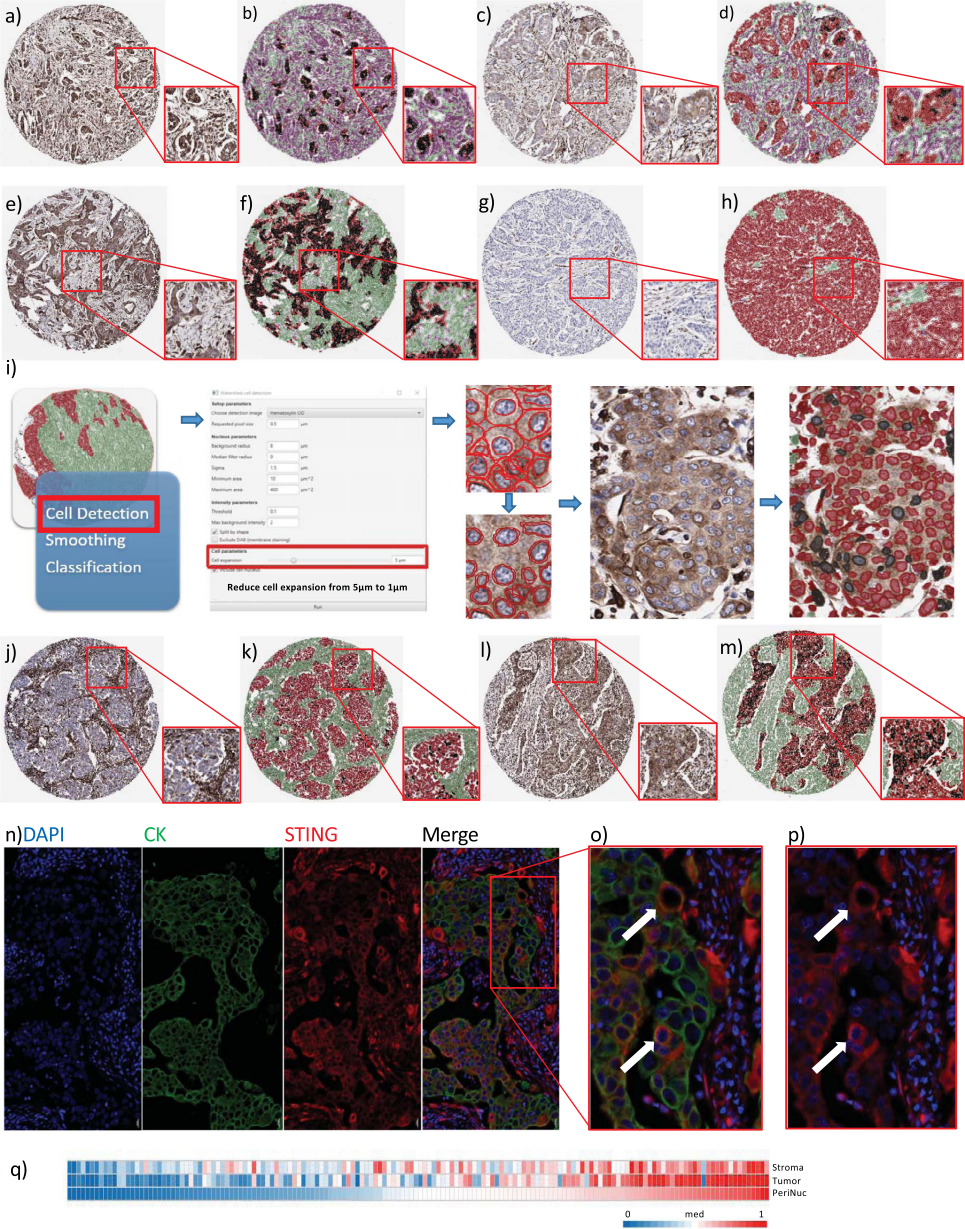
STING immunohistochemistry in breast cancer. Immunohistochemistry (IHC) images representing **a** high expression of STING in both tumor and stromal compartments without and **b** with QuPath mask, **c** low expression of STING in tumor with high expression in stroma without and **d** with QuPath mask, **e** high expression of STING in tumor with low expression in stroma without and **f** with QuPath mask and **g** low expression of STING in both tumor and stromal compartments without and **h** with QuPath mask. Magnification: cores × 4, inset × 8. **i** QuPath workflow for perinuclear STING analysis. Black = perinuclear STING positive cells, Red = perinuclear STING negative cells. IHC images representing **j** low but detectable perinuclear STING in an otherwise STING positive tumor without and **k** with QuPath mask and **l** high perinuclear STING expression without and **m** with QuPath mask. Magnification: cores x 4, inset x 8. **n** Multiplex IHC of tumor section with DAPI, STING (red) and cytokeratin (CK, green). Co-localization of STING and CK is demonstrated in **o** (with CK) and **p** (without CK), indicated by the white arrows. Magnification: **n** ×10, **o**, **p** ×20. **q** Correlation of stromal, tumor and perinuclear STING (absolute scores measured as percentage of positive cells) in breast cancer IHC cases. Stromal v. tumor: *R* = 0.7240, *p* < 0.0001, Stromal v. perinuclear: *R* = 0.6916, *p* < 0.0001, Tumor v. perinuclear: *R* = 0.8496, *p* < 0.0001.

To address the role of STING activation and relationship with the tumor immune microenvironment, we utilized a previously described high risk, early stage breast tumor TMA where individuals were all treated surgically followed by adjuvant anthracycline-based chemotherapy.^15,16^ Following review for quality control, STING IHC was available for 156 tumors (clinicopathological characteristics in Supplementary Table 1). We first assessed the expression of STING in a subset of tumor cores. Four distinct patterns of STING expression were observed (Fig. 1a–h, Supplementary Fig. 1a, b), where STING expression was high throughout the tumor, high in stromal or tumor epithelial compartment alone, or universally low/absent. In tumors with epithelial cell STING expression, we noted a proportion of cells with a distinctive perinuclear pattern of STING staining. As STING migrates to perinuclear microsomes when activated^17^ (Supplementary Fig. 1c), we measured perinuclear STING expression as representative of tumor cell STING activity. Therefore, we applied a digital pathology workflow (Fig. 1i) to calculate perinuclear STING (pnSTING) expression in the DAB IHC digital images, where pnSTING was defined as within 1 μm of the nuclear membrane (as described above). Within STING positive tumors, we were able to determine tumors with low (Fig. 1j, k) or high proportions of perinuclear STING cells (Fig. 1l, m).

To investigate whether observed pnSTING staining was found in tumor epithelial cells or infiltrating immune cells, we performed multiplex studies on selected sections. Close examination verified that pnSTING staining cells were within cytokeratin (CK) positive tumor nests, confirming these cells were not of immune origin (Fig. 1n–p). Nearly all cases had some level of STING expression within the stroma, with the majority of these also expressing STING within tumor cells (*R* = 0.6916, *p* < 0.0001). While total STING levels within the tumor closely correlated with high pnSTING positive cells (*R* = 0.850, *p* < 0.0001), pnSTING high tumors were a clearly defined subset within STING-expressing tumors (Fig. 1q, Supplementary Fig. 1d).

**Fig. 2:**
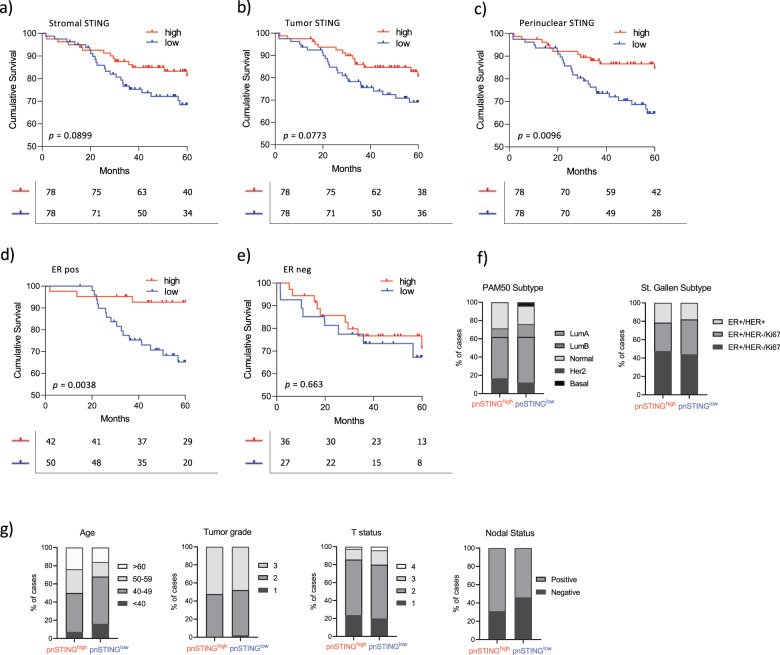
pnSTING IHC score predicts outcome in ER+ breast cancer. Kaplan–Meier Curve of relapse free survival (RFS) stratified based on high (above median) or low (below median) of STING expression in the **a** stromal compartment, **b** tumor epithelial compartment or **c** perinuclear region. Kaplan Meier Curve of relapse free survival (RFS) stratified based on high (above median of all cases) or low (below median of all cases) of STING expression in the perinuclear region in **d** ER positive (ER+) and **e** ER negative (ER-) cases. **f** Stacked bar chart of the percentage of ER + patients stratified based on high (above median) or low (below median) of STING expression in the perinuclear region based PAM50 subtype and St. Gallen subtype. **g** Stacked bar chart of the percentage of ER+ patients stratified based on high or low pnSTING expression detailing clinicopathological characteristics of Age, Tumor Grade, T stage, N status (from left to right).

The percentage of positive pnSTING cells within the tumor (defined as number of positive cells/total number of tumor cells × 100) was delineated by a machine learning digital pathology classifier. High pnSTING (pnSTING^high^) tumors were defined as tumors containing > median pnSTING positive cells for the cohort, as an unbiased classifier. Using this cut-off, pnSTING^high^ predicted significantly improved relapse free survival (RFS) (HR = 0.405, 95% CI 0.210–0.778, *p* = 0.0096) compared to either stromal or whole cell tumor STING expression alone (HR = 0.553, 95% CI 0.29–1.05, *p* = 0.090; HR = 0.554, 95% CI = 0.291–1.06, *p* = 0.077 respectively) (Fig. 2a–c, Supplementary Table 2). However, the predictive ability of pnSTING expression, when patients are split into ER + and ER- groups, was independently significant in the ER + population (multivariate analysis HR = 0.206, 95% CI 0.059–0.727, *p* = 0.014), and did not predict RFS in ER- disease (HR = 0.810, 95% CI 0.309–2.12, *p* = 0.663) (Fig. 2d, e, Supplementary Table 3). It is important to note, however, when we formally tested the effect of the interaction term on ER status using a likelihood ratio test it was not found to be significant (*p* = 0.1229). This may reflect the fact that this cohort only has 7% power to detect a true difference in ER- patients while having a 78% power in the ER + patients (which in of itself was also underpowered). While overall survival was numerically improved in the ER+ pnSTING^high^ subgroup, this was not significant at 60 months (HR = 0.6288, 95% 0.2201–1.796), which may be due to the relatively short follow-up (Supplementary Fig. 2a-e). Using consensus breast subgroups^18^ pnSTING^high^ predicted RFS in the Luminal A (ER + , HER2−, Ki67−) subgroup (HR = 0.130, 95% CI = 0.002–0.757, *p* = 0.0232) (Supplementary Fig. 2f–m, Supplementary Table 4). Comparing pnSTING^high^ and pnSTING^low^ ER + cases, no significant difference in PAM50 subtype, St. Gallen subtype, grade, age, T or N status or treatment received was observed (Fig. 2f, g, Supplementary Fig. 2n).

Exploration of stromal pnSTING expression proved challenging due to the morphology of stromal cells. In cases with stromal STING expression (Supplementary Fig. 3a–c) staining was intense throughout individual stromal cells, with no clear perinuclear localization of STING expression, in contrast to that observed in tumor epithelial cells. Analysis of gene expression data for predicted fibroblast infiltration^19^ did not identify an association of fibroblasts with pnSTING categories (Supplementary Fig. 3d), although a trend to increased fibroblasts in ER- pnSTING^low^ disease was noted (*p* = 0.0750), which was reversed in ER+ disease (*p* = 0.0895).

### Perinuclear STING expression correlates with the immune landscape of breast cancer

**Fig. 3:**
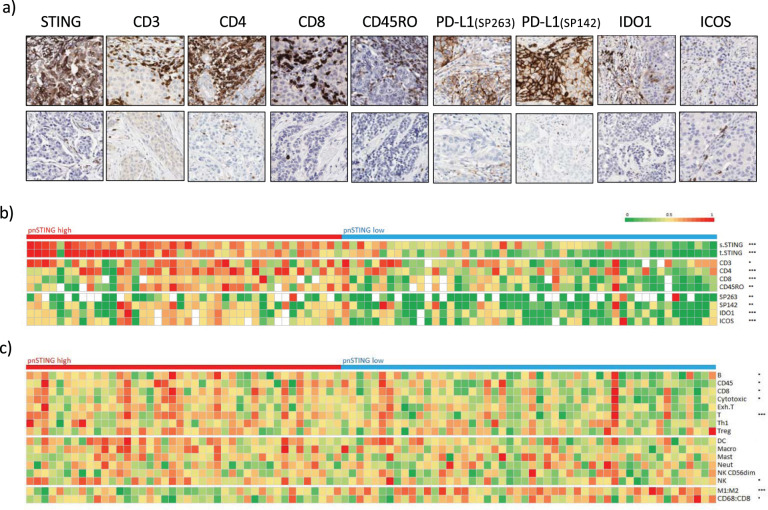
pnSTING and immune correlates in ER + breast cancer. **a** Representative IHC images showing high (top panel) and low (lower panel) expression of perinuclear STING, CD3, CD4, CD8, CD45RO, PD-L1 (SP263), PD-L1 (SP142), IDO1 and ICOS. **b** Heatmap of normalized expression measured by IHC of perinuclear STING, stromal STING, tumor STING, CD3, CD4, CD8, CD45RO, PD-L1 measured by SP263 and SP142, IDO1 and ICOS in ER + breast cancer cases. **c** Heatmap of normalized immune scores derived from deconvolution of microarray data in ER+ breast cancer cases. Correlation between markers and pnSTING stratified based on high (above median) and low (below median) was assessed using the Krushall Wallis test on non-transformed data with *, **, and *** indicating a *p* values of <0.05, <0.01, and <0.001, respectively.

Using matched IHC and array-based gene expression profiling, we characterized the immune landscape in relation to pnSTING expression in both ER + and ER− tumors. Using digital pathological analysis, we measured T- and B-cell markers, innate immune populations and immune checkpoint expression within tumor and surrounding stroma. In ER + cases, a significant association of CD3^+^, CD4^+^, CD8^+^ and CD45RO^+^ cells was identified with pnSTING^high^ tumors (Fig. 3a, b, Supplementary Fig. 4a, Supplementary Table 5). Intratumoral infiltration of CD4^+^, CD8^+^ and CD45RO^+^ was noted in particular, with no significant association of FOXP3^+^ T-regs with pnSTING. In addition, expression of immune checkpoints PD-L1 (clones SP263 and SP142), IDO1 and ICOS significantly correlated with pnSTING^high^ tumors. Surprisingly, in ER- cases, pnSTING did not significantly correlate with T cell markers, suggesting an uncoupling of tumor cell STING activity and immune responses in ER- breast cancer. However, an increase in CD68^+^ and CD163^+^ macrophages was noted in ER- pnSTING^high^ tumors compared to pnSTING^low^ although this was not significant when adjusted for multiple testing (Supplementary Fig. 4b). This trend was not observed in ER + tumors.

**Fig. 4:**
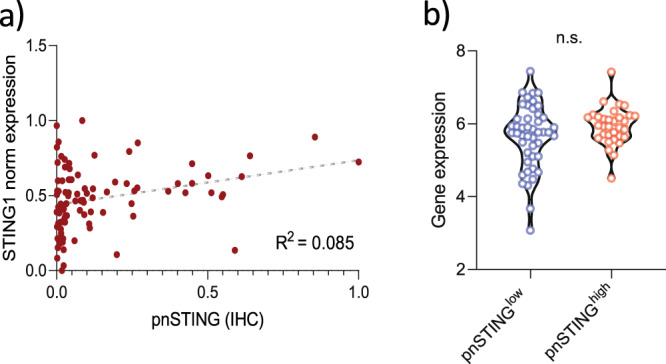
pnSTING and STING1 gene expression. **a** Correlation plot of normalized pnSTING score (IHC) and STING1 gene expression within discovery dataset. *R* = 0.085. **b** Gene expression of STING1 compared between pnSTING^low^ (<median) and pnSTING^high^ (>median) ER + breast cancers. n.s. non significant.

Using gene expression signatures for predicted immune cell infiltration,^20^ we confirmed significant association of similar lymphocytic populations in the TME to that identified on IHC analysis (Fig. 3c, Supplementary Table 6). Dendritic cell (DC) and natural killer cell infiltration (using gene expression scores as IHC was not available for these cell types) were found to be significantly associated with pnSTING^high^ tumors, in keeping with dendritic cells as a key source of PD-L1 and in promoting T-cell responses although the association with DC was not significant when adjusted for multiple testing.^21^ Importantly, gene expression of STING did not strongly correlate with pnSTING scores in ER + tumors (Fig. 4).

In keeping with the IHC data, no significant associations between predicted immune populations from gene expression scores and pnSTING expression were identified in ER- tumors (Supplementary Fig. 4c). However, in ER + tumors, using two independent methods of predicting macrophage polarization,^22,23^ macrophages within pnSTING^low^ tumors were predicted to be “M2”-like or alternatively activated, suggesting an infiltrating pro-tumorigenic myeloid population (Fig. 3c). This is consistent with reports that activation of STING signaling improves activated: inhibitory ratios of tumor-associated macrophages, resulting in an anti-tumor immune response.^24^

### Identification of a gene signature characterizing pnSTING^low^ ER+ poor prognosis tumors

The poor outcomes in pnSTING^low^ ER+ tumors, a typically good prognosis subtype of breast cancer, led us to explore the molecular characteristics defining this subgroup. To characterize pnSTING^high^ and pnSTING^low^ ER + tumors, we performed GSEA which identified a subset of genes enriched in both (Supplementary Table 7). As the genes associated with pnSTING^high^ were predominately immune related and likely derived from the immune-infiltrated TME, we chose to focus on the genes upregulated in pnSTING^low^. Using the top 25 genes altered in pnSTING^low^ tumors, stratification based on high or low gene signature (> median<), replicated the survival differences observed when tumors were stratified based on high and low perinuclear STING IHC expression (Supplementary Fig. 5a–c). To validate this further, we interrogated four independent datasets using the pnSTING^low^ signature. These included: independent samples from the discovery cohort that were not available for IHC-based STING analysis with mRNA expression data available, METABRIC, TCGA 2012, and Wang.^25–27^

**Fig. 5:**
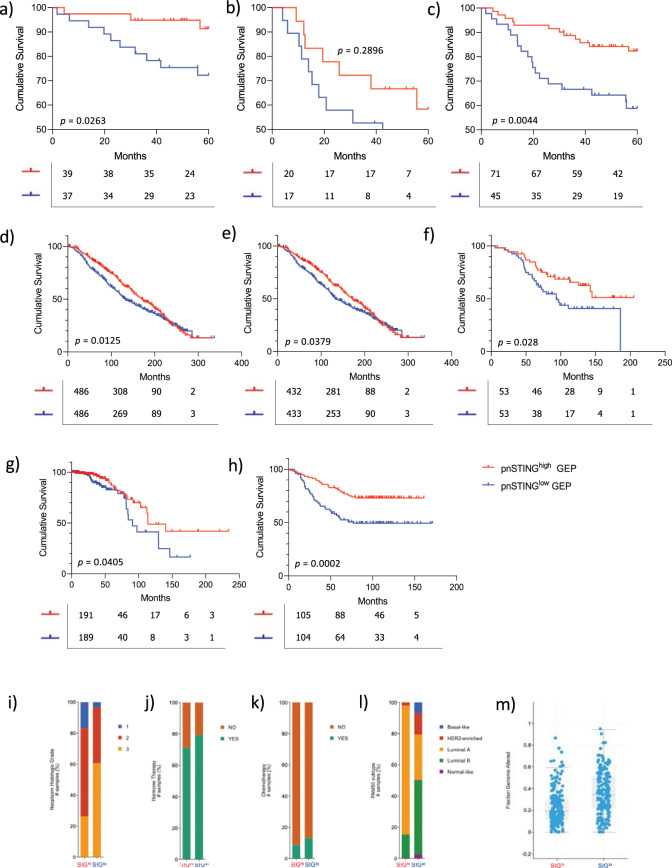
pnSTING^low^ gene expression signature is prognostic of poor outcome in independent datasets. Kaplan Meier Curve analysis of relapse free survival (RFS; months) in **a** ER positive (ER + ), **b** ER negative (ER-) and **c** all samples with gene expression data only from discovery dataset stratified by pnSTING signature score threshold of the IHC cohort. Kaplan Meier Curve analysis of overall survival (months) in **d** ER + disease (all treatments) **e** those receiving hormone therapy only **f** those receiving chemotherapy in the METABRIC dataset stratified by pnSTING signature score. Kaplan Meier Curve analysis of overall survival (months) in ER+ disease from **g** TCGA 2012 dataset and **h** Wang dataset stratified by pnSTING signature score. Clinicopathological and molecular characteristics of METABRIC samples classified by pnSTING gene expression score comparing. **i** Tumor grade (adj. *p* < 0.0001). **j** Hormone therapy (adj. *p* = 0.009). **k** Chemotherapy (adj. *p* = 0.048). Clinicopathological and molecular characteristics of TCGA samples classified by pnSTING gene expression score comparing. **l** PAM50 subtype (adj. *p* < 0.0001) and **m** Fraction genome altered (adj. *p* < 0.0001).

Consistently in each independent dataset, we found the pnSTING^low^ signature predicted poor survival in ER + cases (Fig. 5a–h, Supplementary Table 8). Indeed, with longer follow up data available in the METABRIC dataset, we found that the pnSTING signature significantly predicted overall survival, building on the data obtained in the discovery dataset. Exploring clinicopathological and genomic correlates within the METABRIC and TCGA datasets, ER + pnSTING^low^-signature cases were associated with increased tumor grade (Fig. 5i–k) as well as Luminal B-like cases (Fig. 5l). Increased chromosomal instability, as measured by copy number gain and fraction genome altered, was associated with pnSTING^low^-signature cases (Fig. 5m, Supplementary Fig. 6m). Other significant variations are reported in Supplementary Fig. 6. Importantly, although all samples in the discovery dataset received chemotherapy treatment, suggesting a higher risk population, within the METABRIC cohort we identified little variation in rates of chemotherapy in pnSTING^low^-signature cases (Fig. 5k). This was confirmed in the Wang dataset, where all patients received hormone therapy without chemotherapy. Therefore, we have identified a signature of poor prognosis in ER+ breast cancer independent of systemic anti-cancer therapy received, suggesting alternative approaches should be considered in this subgroup.

### Targetable pathways within pnSTING^low^ poor prognosis tumors

**Fig. 6:**
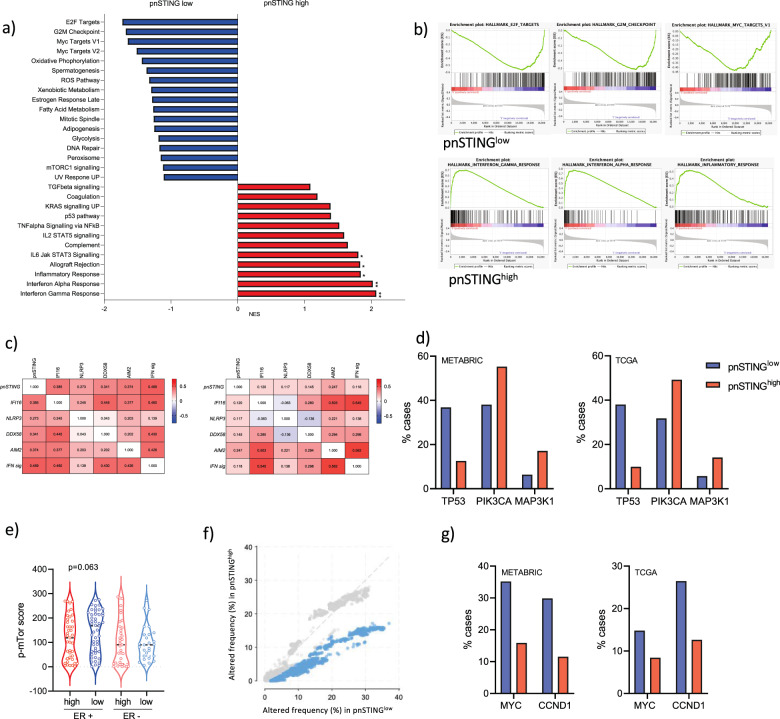
Genomic and transcriptomic analysis of pnSTING signature classified ER + tumors. **a** GSEA results showing Normalized Enrichment Scores (NES) for the gensets enriched >±1 in pnSTING^high^ signature (red) or pnSTING^low^ signature (blue) samples. **b** Enrichment plots for the top 3 genesets enriched in pnSTING^high^ signature or pnSTING^low^ signature samples. **c** Correlation matrices for (left) ER positive and (right) ER negative breast cancer for pnSTING value, gene expression of DNA and RNA sensors and 18-gene interferon signature. **d** Percentage of ER+ cases with mutations in *TP53*, *PIK3CA* and *MAP3K1* in the METABRIC (left) and TCGA (right) 2012 datasets stratified based on high (above median) and low (below median) STING signature score. **e** Expression of p-mTOR (Ser2448) quantified using QuPath in the discovery dataset stratified based on ER expression and perinuclear STING expression (where red = pnSTING^high^ and blue = pnSTING^low^). Percentage of ER+ cases with copy number alteration **f** overall (with significance indicated in blue) in the METABRIC dataset and **g** of *MYC* and *CCND1* in the METABRIC and TCGA 2012 datasets stratified based on high (above median) and low (below median) STING signature score.

Given the consistently poor outcomes observed in ER + pnSTING^low^ breast cancer, we sought to identify potentially targetable pathways. To do so, we utilized multi-omic data in the discovery and validation cohorts. We noted that ER + pnSTING^low^ tumors were associated with genesets associated with chromosomal instability (Fig. 6a, b). Further examination of the individual genes within the signature revealed upregulation of chromatin regulation and DNA repair genes *CDT1*, *NCAPD3,* and *EXO1*. The top gene in this signature, *IMPA2*, has recently been reported to drive cervical cancer progression with a potential novel role in DNA repair.^28^

In contrast to ER + breast cancer, there was an unexpected disconnection between pnSTING and immune cell infiltration in ER- disease, as well as reversal in the prognostic ability of the pnSTING^low^ signature in independent ER- datasets (Supplementary Fig. 5d–f). We analyzed the correlation between pnSTING score (based on IHC data) and gene expression of other cytosolic DNA sensors, as well as an 18-gene T-cell inflamed signature identifying active interferon responses^29^ (Fig. 6c, Supplementary Fig. 7a, b). Importantly, while we observed an expected correlation between STING, other nucleic acid sensors and interferon signaling in ER+ disease, this was notably absent in ER- disease, with no significant correlation observed between pnSTING and interferon activity. As STING has important non-interferon related functions,^30,31^ we hypothesize that post-translational modifications of STING may result in immune-independent functions being the dominant role of STING in ER- breast cancer.

Using the available somatic mutation and copy number alteration data associated with repressed STING signaling in the ER + METABRIC and TCGA 2012 cohorts, we identified high rates of *TP53* mutations (36.8% vs 12.6%; 38.0% vs 9.95% respectively) (Fig. 6d, Supplementary Table 9). Interestingly, a recent report identifies suppression of STING signaling via mutant p53 binding and inhibition of TBK1 downstream of STING in breast cancer cells, suggesting that STING-mediated interferon responses may be prevented in p53 mutant cancer cells via this mechanism.^32^ In contrast, higher rates of mutation in *PI3K* and *MAPK1* were observed in pnSTING^high^ signature cases. *PI3K* mutations have been reported to correlate with Luminal A cancers, and good prognosis, consistent with our data.^33^ In the same report, mTOR pathway activation was associated with Luminal B subtype and poor outcomes. Given that GSEA also identified increased mTOR signaling in pnSTING^low^ samples, we sought to validate this finding by IHC analysis of phosphorylated mTOR in the discovery dataset (Supplementary Fig. 7c-f). Although not significant, a trend towards increased levels of p-mTOR in the pnSTING^low^ cohort was noted, consistent with GSEA (*p* = 0.063, Fig. 6e). mTOR inhibitors have previously been studied in ER + breast cancer, but have lacked a specific biomarker predicting response, and further study of this pathway in pnSTING^low^ tumors may indicate a means of selecting patients for this therapy.

Rates of copy number alteration were significantly increased in pnSTING^low^-signature ER + cases in the METABRIC dataset, although this did not reach significance in the TCGA dataset (Fig. 6f, Supplementary Fig. 7g). In particular, regions encoding *MYC* (8q24) and *CCND1* (11q13) were significantly amplified in pnSTING^low^ cases in both cohorts (Fig. 6g, Supplementary Table 10). *CCDN1* amplification, encoding CyclinD1, results in chromosomal instability via *CDT1,*^34^ consistent with our gene expression findings, suggesting that CDK4/6 inhibitors may also have a role for the treatment of this subgroup of breast cancer. *MYC* amplification was consistent with GSEA identification of upregulation of MYC targets in pnSTING^low^ tumors. Via the ENCODE database, we identified predicted binding sites for MYC and the co-factor MAX (Myc-associated factor X) within *STING* (Supplementary Fig. 8), although it is not known whether MYC directly regulates STING or indirectly regulates downstream responses.

## Discussion

Understanding the regulation of STING-induced immune responses is crucial to understanding immune evasion and improving the anti-cancer effectiveness of immune-targeting and other therapies. By developing a novel digital pathology approach assessing STING on IHC sections, and subsequently identifying a gene signature to classify pnSTING^high/low^ cancers, we were able to validate our finding of poor prognosis in pnSTING^low^ ER + breast cancer in both chemotherapy and hormone therapy-treated tumors. Interestingly, while there was an observed connection between pnSTING and immune infiltration in ER + breast cancers, this was not the case in ER- disease. Previous reports have identified the role of tumor infiltrating lymphocytes in predicting responses to chemotherapy in triple negative and HER2 positive breast cancers, but the role in ER + disease has been less well-defined.^35,36^ This suggests that measuring pnSTING may be able to add granularity to the activation state of the immune infiltrate in ER + disease, therefore identifying tumors with immune restriction and poor clinical outcomes to standard of care, suggesting alternative treatment options should be considered.

Importantly we identified *MYC* amplification associated with chromsomal instability as a potential mechanism of STING repression. Typically, *MYC* is amplified in 15% of breast cancers, and associated with resistance to hormone therapy in Luminal A cancers.^37^ We found *MYC* amplification in up to 35% of pnSTING^low^ ER + breast cancers, a significant enrichment in this subgroup. Amplification of *MYC* is commonly associated with, and a driver of, chromosomal instability.^38^ As chromosomal instability results in cGAS stimulation via micronuclei and the subsequent production of 2′3′cGAMP, it is logical that cancer cells will develop mechanisms to repress anti-cancer immune responses induced by STING. Interestingly MYC has recently been reported to suppress STING signaling in *TP53*- and *BRCA1*-mutant breast cancer.^39^ This supports the hypothesis that amplification of *MYC* is a mechanism of STING repression (and subsequent immunosuppression) in pnSTING^low^ tumors, potentially targetable using novel MYC inhibitors. Indeed, MYC complexes bind directly to IRF5, IRF7, STAT1 and STAT2 in pancreatic cancer, repressing interferon responses.^40^ MYC may have a dual role in cancer progression, promoting both chromosomal instability and direct or indirect suppression of STING-mediated immune responses, resulting in an immunosuppressed microenvironment. The use of novel MYC inhibitors has been shown to increase immune infiltration in preclinical models although the mechanisms driving this infiltrate have not yet been described, and may be STING related.^41^ Combining MYC inhibition and STING activation requires further preclinical study and could be a therapeutic approach in pnSTING^low^ cases.

Other genomic alterations identified in pnSTING^low^ ER + cases include *CCND1* amplification. There are early reports linking CCND1 amplification to immunosuppressive signaling and resistance to ICB.^42^ Interestingly CDK4/6 inhibitors synergize with ICB to enhance responses,^43^ an approach which is now being tested in early phase clinical trials. Another member of the cyclin family, Cyclin E1, is amplified in triple negative breast cancers which have high levels of replicative stress, in keeping with the findings of genomic instability in this poor prognosis ER + breast cancer subgroup.^44^ In addition, the finding of increased mTOR activity in the pnSTING^low^ subgroup may suggest an alternative therapeutic approach. mTOR has complex and varying roles in the TME, including “M2” macrophage polarization and promoting myeloid derived suppressor cell accumulation via upregulation of G-CSF.^45^ In keeping with this, mTOR inhibition demonstrates increased activation and persistence of intratumoral T-cells.^46^

Our study focused on primary/non-metastatic breast cancer, and further work is needed to determine if this chromosomally unstable pnSTING^low^ subgroup persists through metastatic disease. Moreover, we did not identify a clear link between pnSTING and ER status and outcomes in this disease. We suspect that this could be due to the power limitations of our study (7% in ER- and 78% in ER+). ER- breast cancer more commonly involves mutation of DNA repair genes including *BRCA1/2*, *PALB2* and members of the Fanconi Anemia pathway, which result in STING pathway activation. While these DNA repair defects result in aggressive disease, they are vulnerable to DNA-damaging chemotherapy which is standard in the treatment of triple negative breast cancer. This paradox, as well as inherent differences in biology between ER+ and ER- disease, may also contribute to the conflicting findings in ER+ and ER- breast cancer, although further study is needed to provide larger sample sizes as the discovery cohort used in this study is underpowered to assess this fully and so we cannot rule out an association between pnSTING and outcome in ER- disease. However, the fact that, in contrast to ER + disease, we did not observe any significant correlation between pnSTING and immune markers and identified an uncoupling of STING expression and interferon responses in ER- breast cancer suggests that the prognostic role of pnSTING may be more specific to ER + disease. The reasons for this are currently unclear – it may be that STING-modulating mechanisms such as post-translational modifications drive STING towards non-interferon signaling that promotes cancer progression in ER- disease.

While we observed STING staining in the stroma of tumors, we were unable to delineate expression in fibroblasts v. immune cells, for example. Recently, specific efflux and/or influx channels for 2′3′cGAMP have been identified for monocytes, macrophages, fibroblasts and endothelial cells.^47–51^ As STING activation within host immune cells is key for anti-cancer responses, expression of these channels within cancers may drastically alter the nature of STING responses in response to either endogenous or exogeneous STING activation. Expression of STING within the stroma may reflect presence or absence of these transport channels, and further modulate immune responses. Whether these channels differ between ER + and ER- cancers is currently not known. Moreover, cGAS-STING pathway activation within fibroblasts can promote resistance to oncolytic viral therapy, with sensitivity restored by TBK1 inhibition, suggesting STING signaling within fibroblasts may indeed promote tumor progression.^52^ Further exploration of this may address the unexpecting finding of a disconnection between STING and interferon responses in ER- disease.

In summary, taking a novel digital pathology IHC-based approach, we can identify tumors with high intrinsic STING activation. Taken together with identification of immune infiltration, this may identify tumors which respond favorably to immune checkpoint blockade and/or STING agonists. Further exploration of this assay in the context of immune modulating treatment and metastatic disease is warranted. By utilizing multi-omic data from independent breast datasets, we have characterized pnSTING^low^ ER + cancers as a subgroup of breast cancer with intrinsic immunosuppression and chromosomal instability, with poor response rates to standard chemotherapy or endocrine therapy. Further study of pathways resulting in immunosuppression and potential alternative approaches in this subgroup may result in improved patient outcomes.

## Methods

### Cell lines

MDA-MB-436-EV (a kind gift from Paula Haddock, QUB and described in^13^) were maintained in 50% Leibovitz’s L-15/50% RPMI (ThermoFisher Scientific, Paisley, UK) supplemented with 10% fetal bovine serum. Cells were confirmed as mycoplasma free in routine lab testing.

### Immunofluorescence

Cells were seeded on a glass coverslip in 6-well plates and incubated overnight at 37 °C. Cells were then transfected with 10 μg/ml 2′3′cGAMP (Invivogen, Toulouse, France). After 1 h, medium was removed and cells fixed in methanol at −20 °C for 20 min. Slides were covered with a solution of 0.5% (v/v) TritonX-100 in PBS and incubated at room temperature for 15 min. Following 1 h of blocking in 5% (v/v) FBS with 0.2% (v/v) Tween20 in PBS, anti-STING antibody (Cell Signaling Technology, Leiden, the Netherlands) was added at 1:600 and slides incubated at 4 °C overnight. Fluorescently labeled antibody (anti-rabbit IgG, Alexa Fluor488-conjugated, Life Technologies) was added at 1:1500. Following 3 h incubation at room temperature, DAPI was added for 15 min. Slides were mounted using ProLong Gold Antifade Mountant (ThermoFisher Scientific).

### Breast TMA

The cohort of 176 de novo breast cancer patients, their clinical, pathological and outcome parameters and the construction of the tissue microarrays (TMAs) used in the present study has been previously described.^15,16,53^ All identified cases were primary/non-metastatic breast cancer (T1-4, N0-3, M0), resected prior to adjuvant treatment, including both ER + and ER- disease. All patients were treated by surgical resection and adjuvant anthracycline-based chemotherapy as part of standard of care at the time of diagnosis. All ER + patients received hormone therapy and 29/42 HER2 + cases received Herceptin treatment. No ER- patients received hormone therapy and no HER2- patients received Herceptin. Cases were diagnosed in Northern Ireland from 1997 to 2009 with ethical approval granted by the Northern Ireland Biobank^54^ (NIB12-0017, NIB15-0168). STING IHC was approved under NIB19-301. The Northern Ireland Biobank has ethical approval to use de-identified tissue samples from the Belfast Health and Social Care Trust Cellular Pathology archive with matched de-identified data (REC:16/NI/0030). In accordance with the Human Tissue Act, consent is not required for use of archived, de-identified tissue in research studies with ethical approval.

### Immunohistochemistry

All immunohistochemistry (IHC) was performed in a hybrid laboratory (Precision Medicine Centre of Excellence), awarded UK Clinical Pathology Accreditation. Sections were cut from the TMA blocks for H&E staining and IHC for the range of biomarkers described in Supplementary Table 11. Briefly, sections for IHC were cut at 4 μm on a rotary microtome, dried at 37 °C overnight, with IHC then performed on automated immunostainers (Leica Bond-Max, Milton Keynes, UK or Ventana BenchMark, Tucson, AZ). Each biomarker was initially validated on carefully selected control tissues. Antigen-binding sites were detected with a polymer-based detection system (Leica Biosystems UK, Cat. No. DS9800 or Ventana USA Cat. No. 760–700 and Cat. No. 760-500). All sections were subsequently visualized with diaminobenzidine (DAB), counterstained with hematoxylin, and tape mounted using a Sakura Autostainer (Sakura Finetek Europe, Rijn, Netherlands). All slides were scanned on an Aperio AT2 digital scanner (Leica Biosystems, Milton Keynes, UK) at ×40 magnification. Quality control checks ensured images were captured without digital scanning artefacts that might interfere with downstream analysis.

For multiplexing immunofluorescence, 4 μm sections were obtained from cases observed to demonstrate perinuclear STING by DAB IHC. Sections were stained with validated methods, as described.^55,56^ Staining was performed on a Leica Bond-Max (Leica Biosystems UK, Milton Keynes), using Opal 4-Color Automation IHC Kit (CK/STING/DAPI) (Cat. No. NEL820001KT, Akoya Biosciences, Marlborough, MA). According to the manufacturer’s instructions, all retrieval methods and staining steps for Opal were optimized and are detailed in Supplementary Table 11. All multiplex slides were scanned on a Vectra Polaris (Akoya Biosciences) at ×20.

### Image analysis

Digital pathological analysis of all IHC stained TMA slides was performed using QuPath, an open-source image analysis program.^57^ All ×40 scanned slides were imported, dearrayed, and tissue detection carried out to identify the areas of tissue available for cellular analysis. Cores were removed following strict exclusion criteria; e.g., tissue cores that contained <100 tumor cells were removed from analysis. Rigorous quality control steps were taken to remove necrosis, tissue folds, and entrapped normal structures; this was confirmed by a second reviewer with frequent consultation following an established method, previously described.^56,58,59^ For each biomarker, positive staining was defined as the presence of any discernible DAB positivity localized in either the nucleus, membrane and/or cytoplasm depending on the known biological expression. After intensive quality control intensity thresholds were set for cellular DAB detection. Percentage positive data was extracted from each TMA core and averaged across replicates.

A random forest classification method, trained by specific annotations across several cases within a TMA by an experienced image analyst, was employed. Training objects were applied under the supervision of an experienced immunohistochemist prior to analysis. The classifier was trained iteratively via multiple training objects per TMA with between 10 and 20 objects required for subjective acceptance of the classifier to distinguish tumor and stroma compartments. Validation of the classifier was subjectively assessed across the TMAs.

To quantitate pnSTING, adjustment of the default cytoplasm expansion in QuPath was changed from 5 to 1 µm within the tumor class. A digital pathology algorithm was created to capture the objective peri-nuclear staining, subjectively determined by a FRCPath scientist. During the development of the digital pathology process, multiple expansion parameters of the peri-nuclear membrane were assessed to best capture the observed staining pattern. This was further reviewed in many tissue cores across several TMA slides to confirm appropriate definitions before being applied de novo to the whole cohort. A distance of 1 µm was chosen after careful review to best capture the observed pnSTING staining pattern. This resulted in the generation of a pseudo-membrane around the nucleus, which was detected using default parameters in QuPath using the optical density (OD) of hematoxylin. DAB OD mean thresholding was applied within this pseudo-membrane, to differentiate intense pnSTING from that of diffuse cytoplasmic staining.

### Gene expression analysis

As described previously,^15^ total RNA was extracted from macrodissected formalin-fixed paraffin embedded (FFPE) tumor samples using the Roche High Pure RNA Paraffin Kit. Following amplification using the NuGEN WT-Ovation FFPE System (NuGEN, San Carlos, CA), total RNA was hybridized to the Almac Breast Cancer DSA (Affymetrix, Santa Clara, CA).

### Statistical analysis

All statistical analyses were preplanned and corrections for multiple tests applied as appropriate. Correlation was carried out using Spearman test in the context of continuous data or Mann Whitney and Kruskall–Wallis tests where appropriate and *p* values were adjusted using the Benjamini Hochberg method.^60^ Fishers exact or Chi squared analysis were carried out as appropriate using Graphpad Prism v8 Software. Data was transformed for graphing purposes only. In order to define pnSTING high and low cohorts, the R packages “OptimalCutpoints” and “survMisc” were used in analysis. All methods revealed similar values. Therefore, the median value was chosen to dichotomize the data as an unbiased approach.

Heatmaps were generated using robust *z*-score transformed data. Analysis of significance was performed on non-transformed data. Survival analysis was carried out using GraphPad Prism v8 with log rank hazard ratios and p-values reported unless stated otherwise. Multivariate analysis was carried out using the R package “survival” in the context of Age (<40, 40–49, 50–59, >60), T stage (1, 2, 3, 4) and lymph node status (positive or negative). In addition, using this package, the significance of the interaction term was tested by comparing the Cox regression models with or without the interaction term via an ANOVA and a likelihood ratio test. Power analysis was carried out using the R package “Hmisc”.

### Gene Set Enrichment Analysis

Gene Set Enrichment Analysis (GSEA) was carried out using the publicly available GSEA tool (v4.0.3) (www.gsea-msigdb.org) using the Hallmarks gene sets (h.all.v7.1).

### STING activity gene signature

GSEA was used to identify the top 25 ranked genes upregulated in the ER + cases called “STING Perinuclear Low” by IHC analysis. The negative sum of the expression of the 25 genes was calculated from gene expression microarray datasets using gene-level summarizing where appropriate.

### External datasets

Datasets were selected based on the following criteria: Availability of overall and/or progression-free survival data, availability of ER status measured by IHC, availability of microarray mRNA expression data.

The METABRIC^25^ and TCGA Nature 2012 cohorts^26^ were accessed via the cBioPortal website.^61,62^ From METABRIC, the 1338 cases for analysis were selected based on “Breast Invasive Ductal Carcinoma”, availability of ER status measured by IHC and availability of data describing chemotherapy status. For the TCGA Nature 2012 cohort, the 519 cases for analysis were selected based on availability of ER status and mRNA expression data. The Wang et al. dataset^27^ was accessed via GEO datasets (GSE2034) with the associated clinical information used to select ER + cases.

STING gene expression scores were generated. As platforms vary between datasets, it was not possible to apply the same threshold to the METABRIC and TCGA datasets. Therefore, using the median value as an unbiased cut-off, high (above median) and low (below median) scoring tumors were compared using available genomic and clinical information. Of interest, and in support of this approach, the median for the discovery and validation samples from the in-house cohort, which were profiled using the Almac Breast DSA, were very similar (−51.4 and −50.7, respectively). Application of the cut-point from the discovery cohort to the validation cohort results in only one sample being re-classified with significant differences in survival observed also when the median of the validation cohort is applied (RFS: HR 0.2795 (0.094160–0.8295) *p* = 0.0383).

### Reporting summary

Further information on research design is available in the Nature Research Reporting Summary in this article.

## Supplementary information

Supplementary Information

Reporting Summary

## Data Availability

The data generated and analyzed during this study are described in the following data record: 10.6084/m9.figshare.14637759.^63^ Gene expression data matched to the tissue microarray samples from Northern Ireland Biobank (NIB) was described previously in 10.1093/jnci/djt335; the data are openly available in Gene Expression Omnibus under the following accessions: https://identifiers.org/geo:GSE16334,^64^ https://identifiers.org/geo:GSE20271,^65^ https://identifiers.org/geo:GSE22093,^66^ https://identifiers.org/geo:GSE6861,^67^ https://identifiers.org/geo:GSE7390,^68^ https://identifiers.org/geo:GSE2034,^69^ https://identifiers.org/geo:GSE2990.^70^ The METABRIC and TCGA Nature 2012 cohorts were accessed via the cBioPortal website. The Wang et al. dataset was accessed via GEO datasets (GSE2034^69^) with the associated clinical information used to select ER+ cases. The gene set enrichment analysis (GSEA) outputs are stored in the file “my_analysis.Gsea.1590420309857” (GSEA tool (v4.0.3); www.gsea-msigdb.org), housed on institutional storage and available upon request. The files listed below are housed on institutional storage at Queen’s University Belfast and are not openly available as the data contain potentially sensitive patient data and the ethical agreement with NIB did not include permission to share. Collaborations using these files may be considered: contact the corresponding author to inquire. The files are: “STING and Consolidated TMA and GE data.xlsx”, “mTOR H-score Data.xlsx”, and all Breast 300 IHC images.
