# Supplementary material for: The clinical and molecular significance associated with STING signaling in breast cancer
Source: NPJ Breast Cancer. 2021 Jun 25;7:81. doi: 10.1038/s41523-021-00283-z (PMC8233333; doi:10.1038/s41523-021-00283-z)
Supplement: Supplementary file 1 — Supplementary Information [file 41523_2021_283_MOESM1_ESM.pdf]

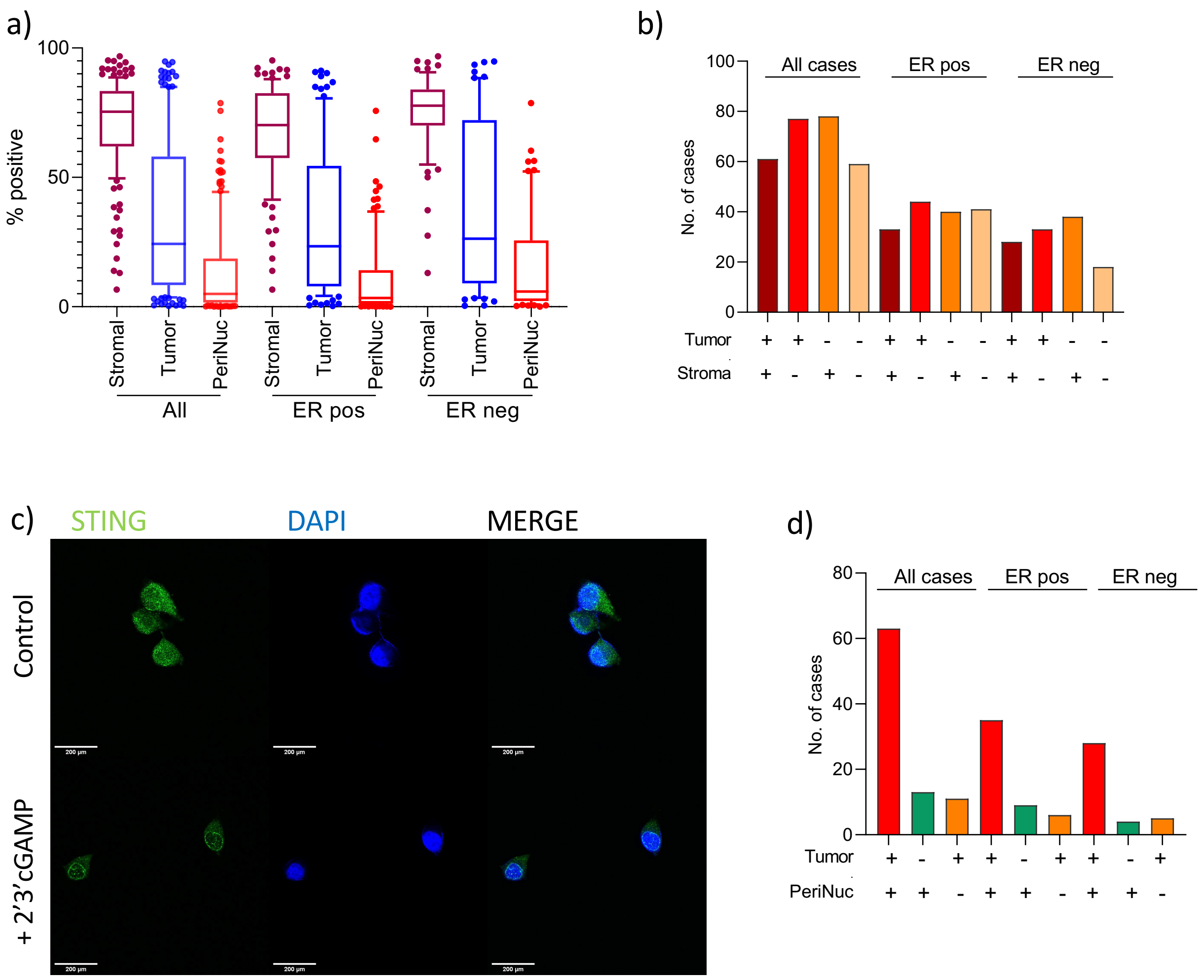

Supplementary Figure 1: Tumor, Stromal and Perinuclear STING expression in breast cancer
a) Percentage positive cells for stromal, tumor and perinuclear STING in ER positive and ER negative tumors in discovery dataset. **b**) Distribution of tumor and stromal expression of STING where + represents > median expression and - represents < median expression. Median is calculated on whole dataset expression. **c**) Immunofluorescence images of MDA436-EV cells treated with either control or 2’3’ cGAMP (10 µg/ml) for 60 minutes. Scale bar = 200 µm. **d**) Unique expression of tumor or perinuclear-only STING in all cases, ER positive and ER negative breast tumors where + represents > median expression and - represents < median expression. Median is calculated on whole dataset expression.

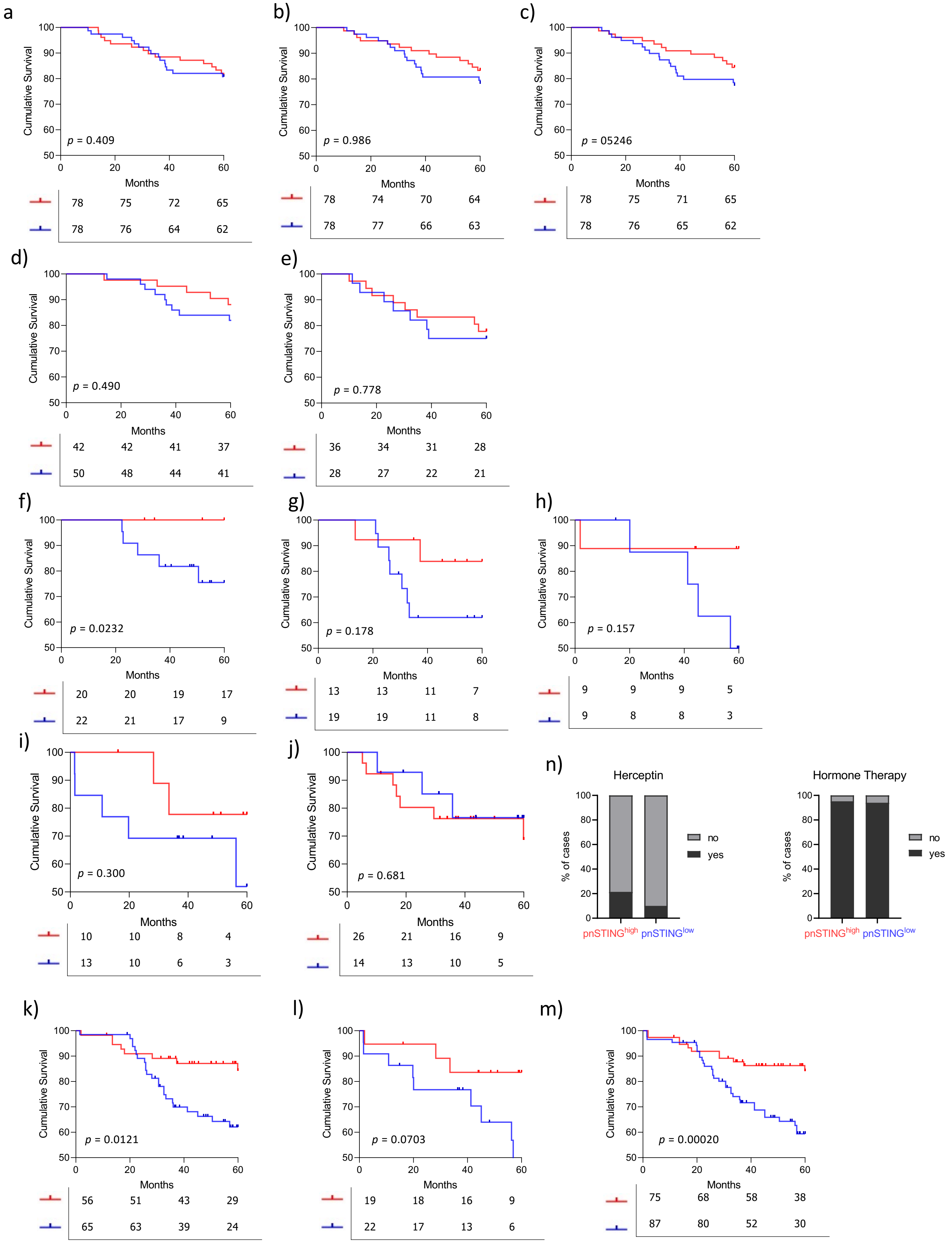
a
p = 0.409
78 75 72 65
78 76 64 62
b)
p = 0.986
78 74 70 64
78 77 66 63
c)
p = 05246
78 75 71 65
78 76 65 62
d)
p = 0.490
42 42 41 37
50 48 44 41
e)
p = 0.778
36 34 31 28
28 27 22 21
f)
p = 0.0232
20 20 19 17
22 21 17 9
g)
p = 0.178
13 13 11 7
19 19 11 8
h)
p = 0.157
9 9 9 5
9 8 8 3
i)
p = 0.300
10 10 8 4
13 10 6 3
j)
p = 0.681
26 21 16 9
14 13 10 5
n)
Herceptin
Hormone Therapy
% of cases
no
yes
pnSTINGhigh
pnSTINGlow
k)
p = 0.0121
56 51 43 29
65 63 39 24
l)
p = 0.0703
19 18 16 9
22 17 13 6
m)
p = 0.00020
75 68 58 38
87 80 52 30
Cumulative Survival
Months

Supplementary Figure 2: pnSTING IHC score predicts outcome in ER+ breast cancer
Kaplan Meier Curve of overall survival (OS) stratified based on high (above median) or low (below median) of STING expression in the **a**) stromal compartment, **b**) tumour epithelial compartment or **c**) perinuclear region. Kaplan Meier Curve of overall survival (OS) stratified based on high (above median of all cases) or low (below median of all cases) of STING expression in the perinuclear region in **d**) ER positive (ER+) and **e**) ER negative (ER-) cases. Kaplan Meier Curve of Relapse Free Survival (RFS) stratified based on high (above median) or low (below median) of STING expression in the perinuclear region in St Gallen subtypes: **f**) ER+/HER2-/Ki67-, **g**) ER+/HER2-/Ki67+, **h**) ER+/HER2+/Ki67+, **i**) ER-/HER2+ and **j**) ER-/PR-/HER2- (TNBC). Kaplan Meier Curve of Relapse Free Survival (RFS) stratified based on high (above median) or low (below median) of STING expression in the perinuclear region in **k**) ER+/HER2-/Ki67+/- , **l**) ER+/- /HER2+and **m**) Non TNBC. **n**) Stacked bar chart of the percentage of ER+ patients stratified based on high (above median) or low (below median) of STING expression in the perinuclear region based on clinical attributes: Herceptin Treatment (left panel), Hormone Therapy (right panel).

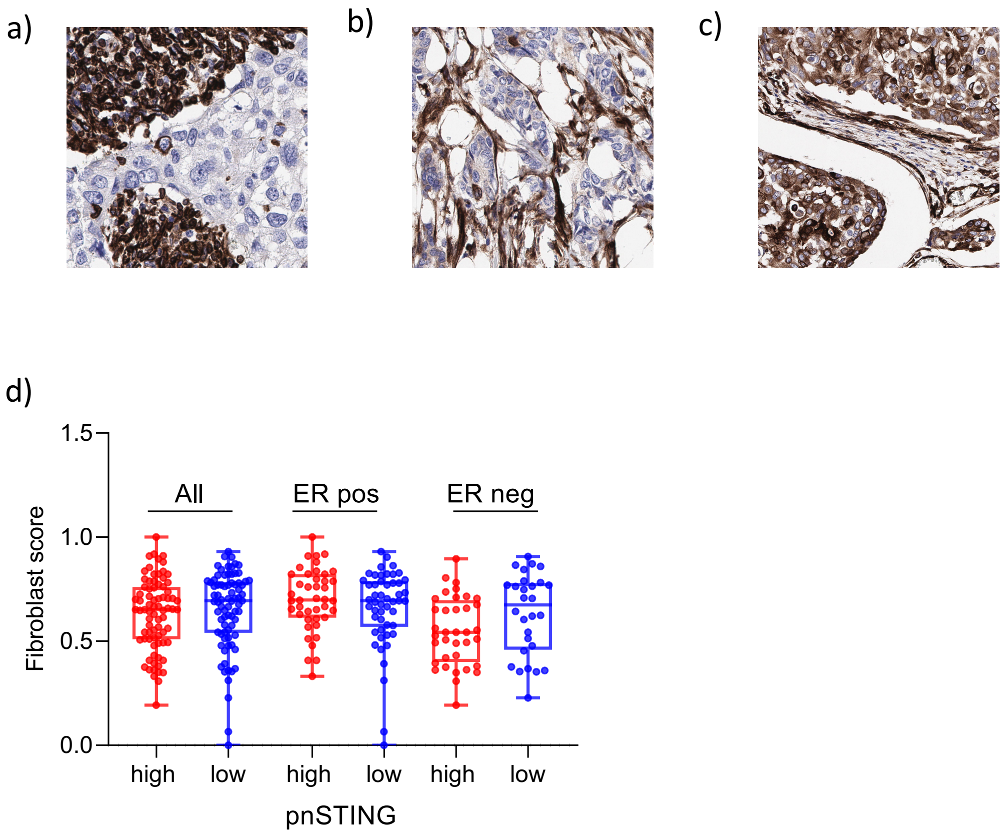

Supplementary Figure 3: Stromal STING expression and association with fibroblasts in the tumor microenvironment

a-c) IHC images of STING staining in samples where stromal regions are positive for STING. Magnification x 40. STING expression cannot be delineated within stromal regions. **d**) Expression of stromal fibroblast gene expression signature in the discovery dataset, ER+ and ER- disease as shown. pnSTINGhigh v low in both: $p = 0.528$; in ER+ $p = 0.0895$; in ER- $p = 0.0750$.

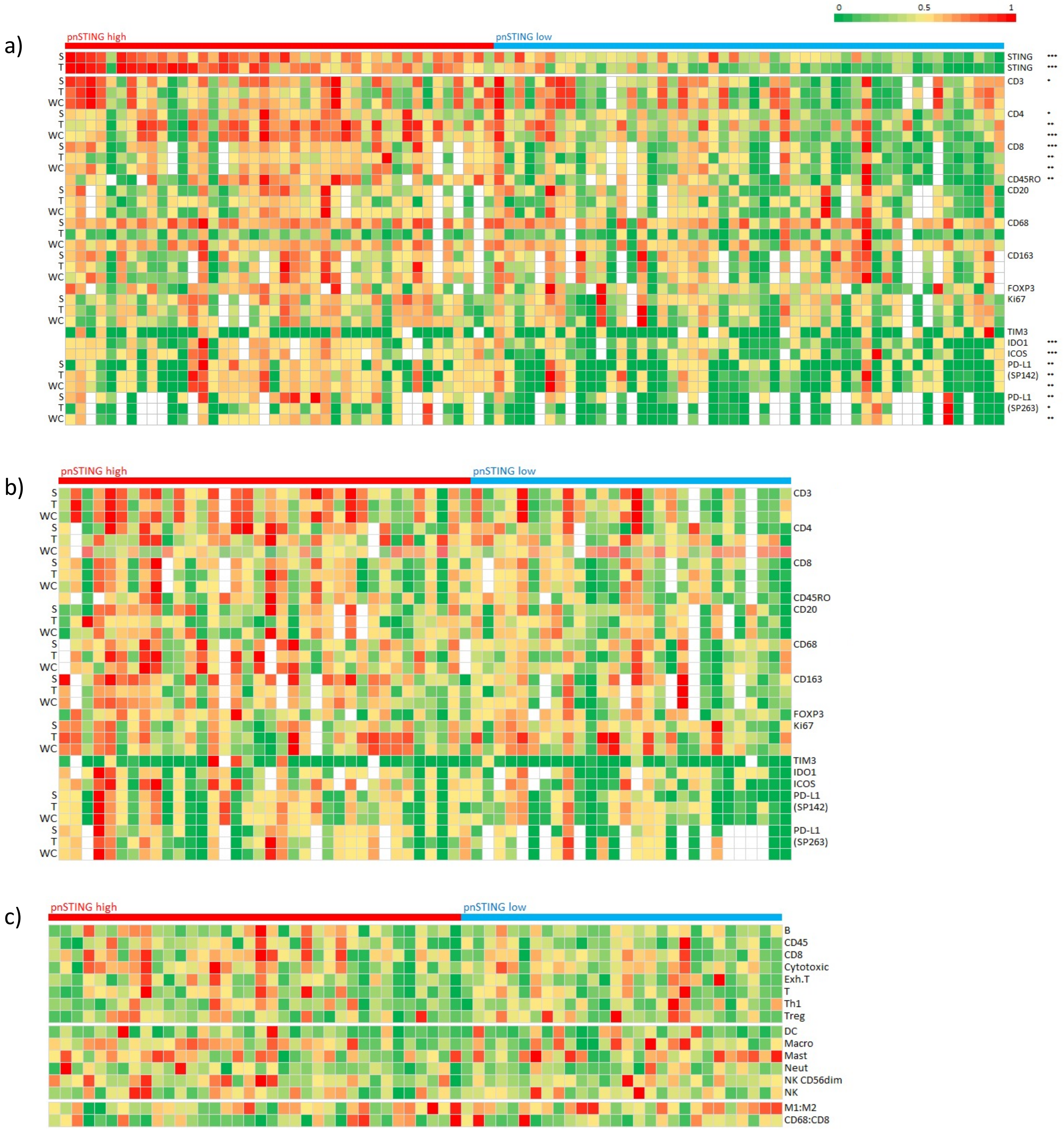
a)
b)
c)
0
0.5
1
pnSTING high
pnSTING low
S
T
WC
STING
CD3
CD4
CD8
CD45RO
CD20
CD68
CD163
FOXP3
Ki67
TIM3
IDO1
ICOS
PD-L1
(SP142)
PD-L1
(SP263)
B
CD45
CD8
Cytotoxic
Exh.T
T
Th1
Treg
DC
Macro
Mast
Neut
NK CD56dim
NK
M1:M2
CD68:CD8

Supplementary Figure 4: pnSTING immune correlates in ER+ and ER- breast cancer

a) Heatmap of normalized expression measured by IHC of perinuclear STING, stroma STING, tumor STING, CD3, CD4, CD68, CD45RO, CD163, FOXP3, CD8, CD20, Ki-67, PD-L1 measured by SP263 and SP142, TIM3, IDO1 and ICOS in ER+ breast cancer cases. Expression was quantified in the stroma compartment (S), tumor epithelial compartment (T) or whole core (WC) as indicated. **b**) Heatmap of normalized expression measured by IHC of perinuclear STING, stroma STING, tumor STING, CD3, CD4, CD68, CD45RO, CD163, FOXP3, CD8, CD20, Ki-67, PD-L1 measured by SP263 and SP142, TIM3, IDO1 and ICOS in ER- breast cancer cases. Expression was quantified in the stroma compartment (S), tumor epithelial compartment (T) or whole core (WC) as indicated. **c**) Heatmap of normalized immune scores derived from deconvolution of microarray data in ER- breast cancer cases. Correlation between markers and perinuclear STING stratified based on high (above median) and low (below median) using the Krushall Wallis test on non-transformed data with *, ** and *** indicating p values of < 0.05, < 0.01 and < 0.001 respectively.

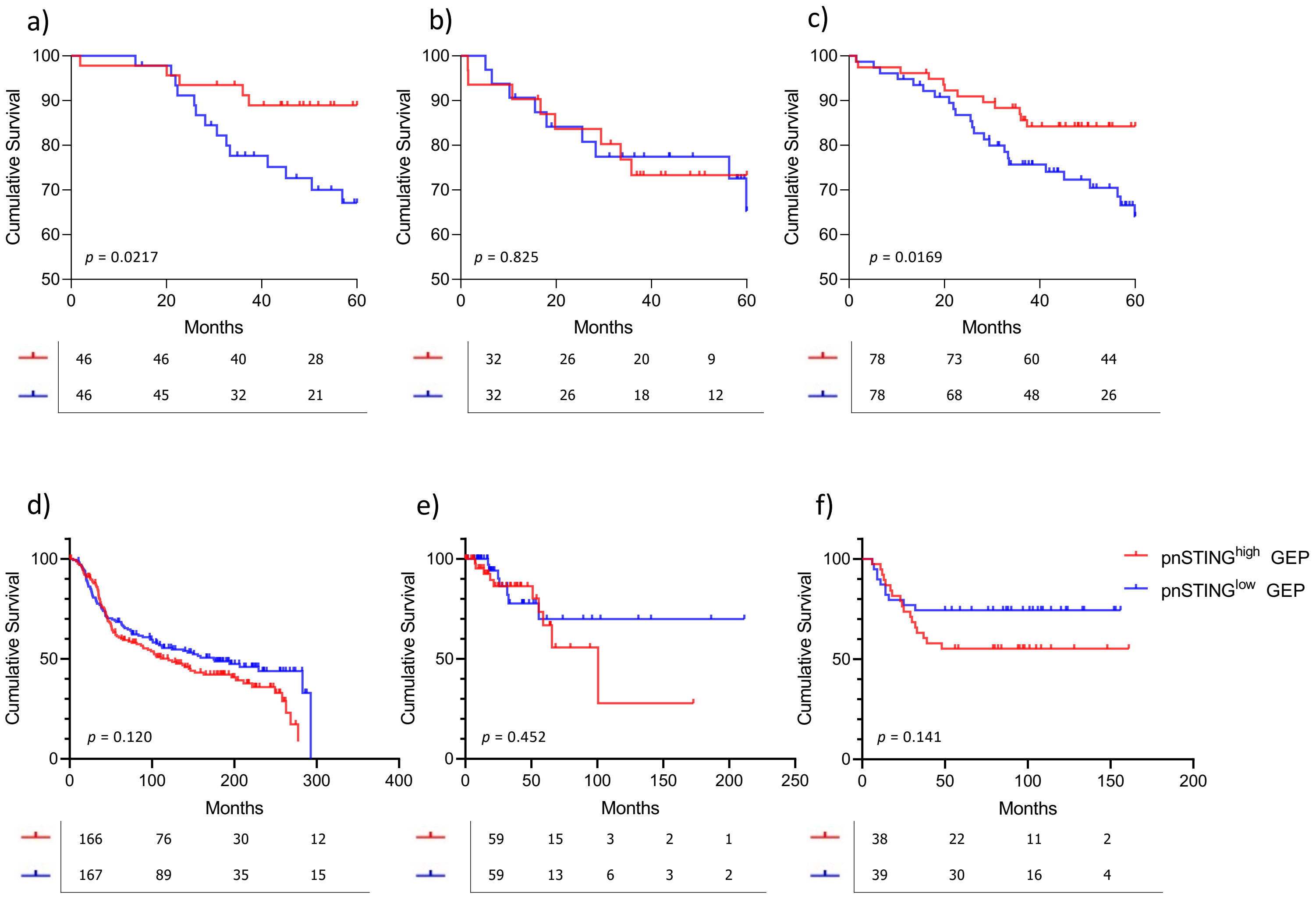

Supplementary Figure 5: pnSTING^{low} gene expression signature in ER negative breast cancer

Kaplan Meier Curve analysis of relapse free survival (RFS - months) in **a**) ER positive (ER+), **b**) ER negative (ER-) and **c**) all samples with STING IHC data from discovery dataset stratified by pnSTING signature score. Kaplan Meier Curve analysis of overall survival (months) in ER- disease from the **d**) METABRIC **e**) TCGA 2012 and **f**) Wang datasets.

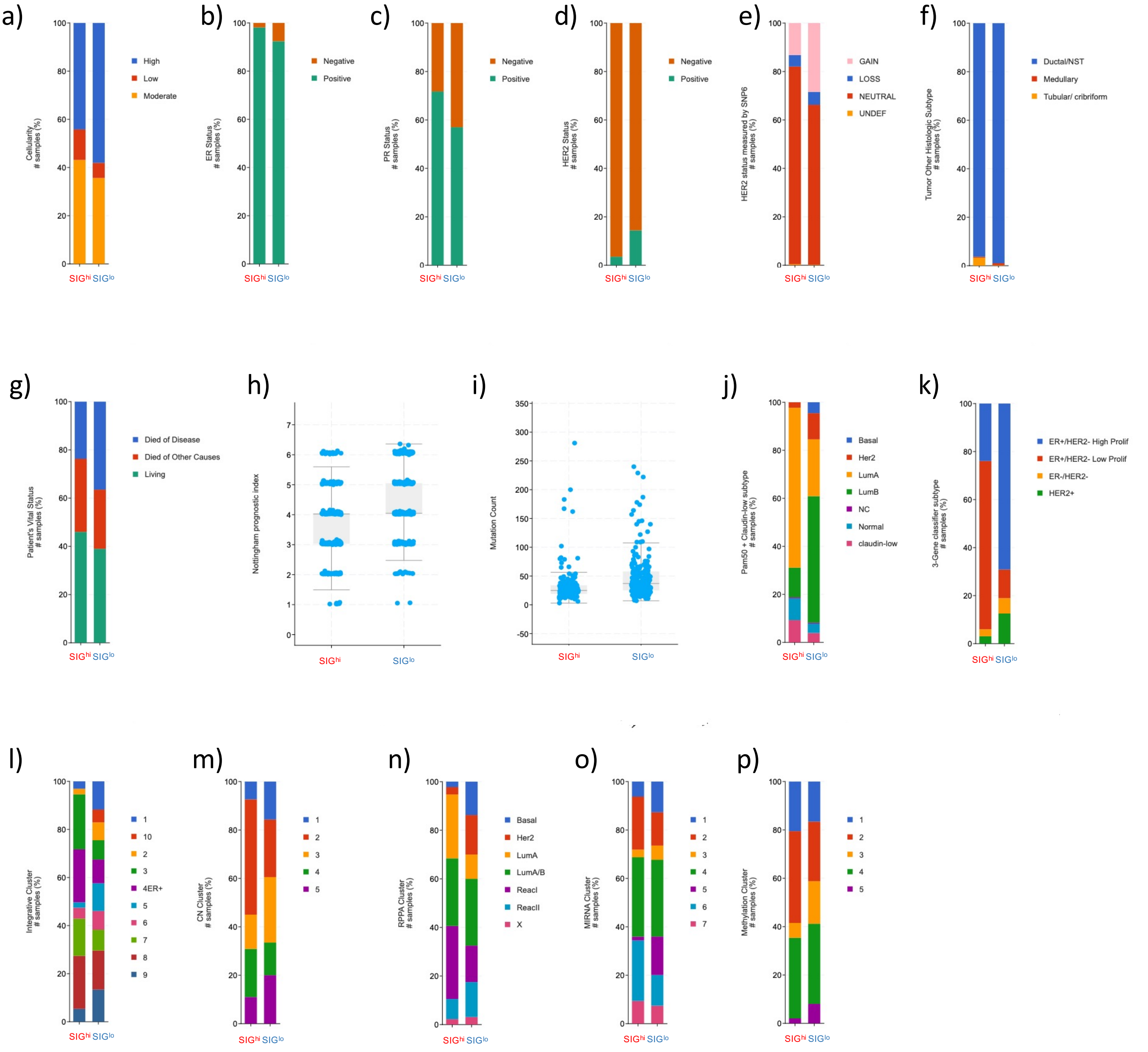

Supplementary Figure 6: Clincopathological and molecular characteristics of pnSTING^{high} and $^{-low}$ breast cancers

METABRIC and TCGA ER+ cohort data stratified by pnSTING signature score for **a**) Cellularity **b**) ER status **c**) PR status **d**) HER2 status **e**) HER2 status by SNP6 measurement **f**) histological subtype. **g**) Patient outcomes. **h**) Nottingham prognostic index score (all METABRIC) **i**) Mutational load (TCGA) **j**) PAM50 and Claudin low classified subgroups **k**) 3-gene classifier (METABRIC) **l**) Integrative Clusters **m**) CN clusters **n**) RPPA **o**) miRNA clusters and **p**) Methylation clusters (TCGA).

a)

	pnSTING	IFI16	NLRP3	DDX58	AIM2	IFN sig
pnSTING		1.51E-04	8.35E-03	8.84E-04	2.41E-04	2.4E-06
IFI16	1.51E-04		1.86E-02	9.00E-06	2.08E-04	3.9E-06
NLRP3	8.35E-03	0.0186		0.685	0.0521	0.186
DDX58	8.84E-04	9.00E-06	0.685		0.0534	1.9E-05
AIM2	2.41E-04	2.08E-04	0.052	0.0534		2.3E-05
IFN sig	2.36E-06	3.89E-06	0.186	1.90E-05	2.25E-05	

b)

	pnSTING	IFI16	NLRP3	DDX58	AIM2	IFN sig
pnSTING		0.344	0.357	0.254	0.0493	0.354
IFI16	0.344		0.619	0.025	2.25E-05	3.24E-06
NLRP3	0.357	0.619		0.285	0.0787	0.278
DDX58	0.254	0.025	0.285		0.0184	0.017
AIM2	0.049	2.25E-05	0.079	0.018		1.38E-06
IFN sig	0.354	3.24E-06	0.278	0.017	1.38E-06	

c) d) e) f)

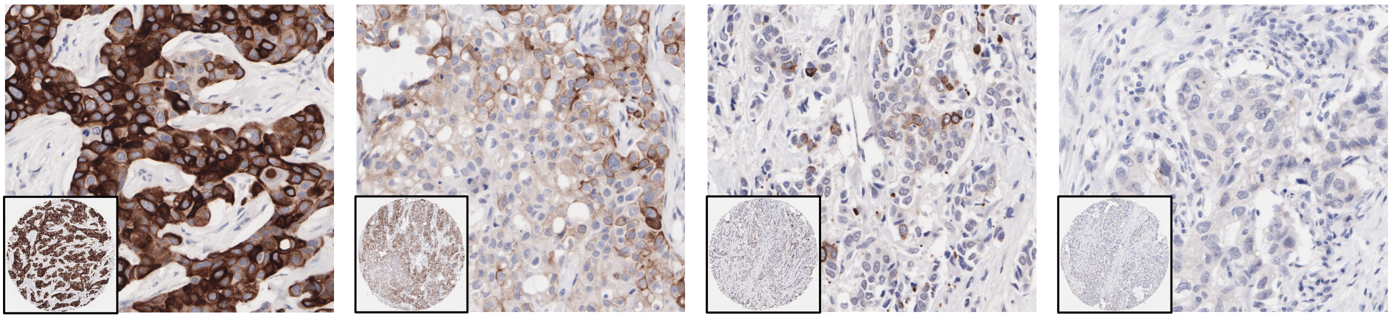

g)

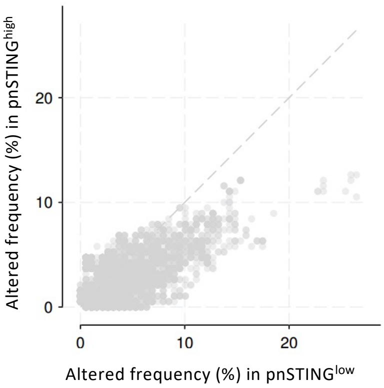

Supplementary Figure 7: mTOR expression and pnSTING correlations

p values for calculated correlations in **a**) ER positive (left table) and **b**) ER negative (right table) breast cancers for pnSTING values, gene expression of cytosolic DNA and RNA sensors and interferon signaling. Non-significant values are indicated in blue. Representative IHC images of breast cancer samples with **c**) high, **d**) medium, **e**) low and **f**) absent mTOR signaling using phospho-specific mTOR antibody (Ser2448). Magnification x 40, Inset x 10. **g**) Percentage of ER+ cases with copy number alteration overall in the TCGA dataset.

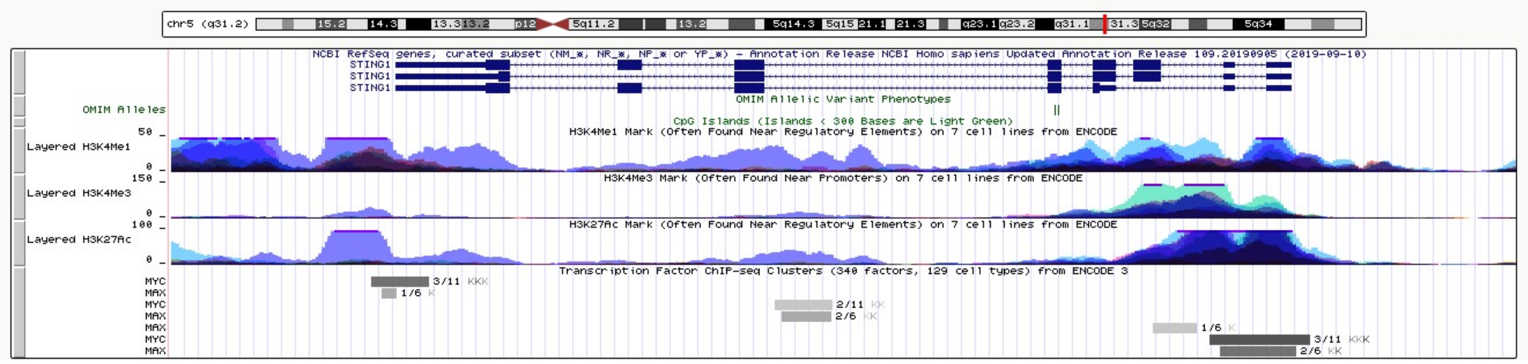

Supplementary Figure 8: Transcription factor analysis of STING1 using ENCODE database
MYC and MAX predicted binding sites on *STING1*. (Note: *STING1* is encoded on the negative strand and therefore is viewed from right to left.)

Supplementary Table 1: Clinicopathological details of pnSTING scored cohort

**Characteristics (N=156)**	**N**	**%**
Tumor Grade		
**G1**	1	0.6
**G2**	51	32.7
**G3**	104	66.7
pN Stage (nodal involvement)		
**N0**	68	43.6
**N1mi**	2	1.3
**N1**	50	32.1
**N3**	21	23.5
**N3**	15	9.5
pT stage		
**T1**	31	19.9
**T2**	93	59.6
**T3**	27	17.3
**T4**	5	3.2
Histological Type		
**Ductal (no special type)**	128	82
**Lobular (no special type)**	14	9
**Mixed ductal and lobular**	11	7.1
**Other**	3	1.9
ER status		
**Positive**	92	59
**Negative**	64	41

ER: Estrogen receptor; pT: pathological tumor stage; pN: pathological lymph node stage.

Supplementary Table 2: STING expression and relapse free survival in discovery cohort (ER+ and ER- breast cancer)

Relapse Free Survival		**N(n)**	**HR**	**95% CI**	**p-value**
		156 (37)		Univariate	
**STING Stroma**	low	78 (23)	1		
	high	78 (14)	0.5528	0.29-1.054	0.0899
**STING Tumor**	low	78 (23)	1		
	high	78 (14)	0.5543	0.2908-1.057	0.0773
**STING Perinuclear**	low	78 (26)	1		
	high	78 (11)	0.4046	0.2104-0.778	0.0096**

HR: Hazard ratio, CI: Confidence interval; n: events

Supplementary Table 3: STING expression and relapse free survival in ER+ and ER- breast cancer

ER+		**N(n)**	**HR**	**95% CI**	**p-value**
Relapse Free Survival		92 (19)		**Univariate**	
**STING Perinuclear**	low	50 (16)	1		
	high	42 (3)	0.1955	0.0795 – 0.4805	0.0038**
				**Multivariate**	
**STING Perinuclear**	low	50 (16)	1		
	high	42 (3)	0.2067	0.0588-0.7265	0.014*
ER-					
Relapse Free Survival		64 (17)		**Univariate**	
**STING Perinuclear**	low	27 (8)	1		
	high	36 (9)	0.8098	0.3089-2.123	0.6633

Multivariate in context of age (<40, 40-49, 50-59, >60), T status (1, 2, 3,4) and Lymph node (pos/neg). No other factor was significant.

Supplementary Table 4: Relapse free survival stratified by pnSTING (low/high) in consensus breast subgroups

		N(n)	HR	95% CI	p-value
Relapse Free Survival				Univariate	
**ER+/HER2-/Ki67-**	low	22 (5)	1		
	high	20 (0)	0.1304#	0.0022-0.7568	0.0232*
**ER+/HER2-/Ki67+**	low	19 (7)	1		
	high	13 (2)	0.3570	0.0959-1.329	0.1776
**ER+/HER2+/Ki67+**	low	9 (4)	1		
	high	9 (1)	0.2347	0.0406-1.356	0.1574
**ER-/HER2+**	low	13 (5)	1		
	high	10 (2)	0.4320	0.0981-1.903	0.3005
**ER-/PR/HER2-**	low	14 (3)	1		
	high	26 (7)	1.327	0.3656-4.818	0.6805
**ER+/HER2-/Ki67+/-**	low	65 (23)	1		
	high	56 (8)	0.3729	0.1843-0.7542	0.0121*
**ER+/-/HER2+**	low	22(9)	1		
	high	19 (3)	0.3196	0.1030-0.9912	0.0703
**Non-ER-/PR-/HER2-**	low	87 (32)	1		
	high	75 (11)	0.3573	0.1965-0.6497	0.0020*

#Mantel-Haenszel HR reported

Supplementary Table 5: Associations of immunohistochemistry immune markers with ER+ and ER- breast cancer

Perinuclear STING high vs low	Compartment	ER+ p-value	ER+ adjusted p-value	Pearson R	ER- p-value	ER- adjusted p-value	Pearson R
**CD3**	Stromal	0.021	0.04	0.261	0.037	0.20	0.201
	Tumoral	0.73	0.73	0.164	0.18	0.36	0.227
	Whole core	0.15	0.25	0.219	0.098	0.26	0.230
**CD4**	Stromal	0.017	0.0036	0.102	0.057	0.26	0.199
	Tumoral	0.00095	0.0028	0.037	0.96	0.96	0.002
	Whole core	0.00012	0.00077	0.136	0.085	0.26	0.182
**CD8**	Stromal	0.000087	0.00077	0.256	0.23	0.38	0.307
	Tumoral	0.00049	0.0020	0.030	0.33	0.46	0.219
	Whole core	0.000091	0.0077	0.180	0.24	0.38	0.272
**CD45RO**		0.00011	0.0077	0.554	0.093	0.26	0.164
**CD20**	Stromal	0.52	0.57	0.412	0.48	0.61	0.125
	Tumoral	0.15	0.25	0.261	0.88	0.92	0.188
	Whole core	0.21	0.28	0.003	0.8	0.88	0.077
**CD68**	Stromal	0.1	0.19	0.085	0.23	0.38	0.202
	Tumoral	0.36	0.43	-0.030	0.008	0.16	0.418
	Whole core	0.24	0.31	0.035	0.064	0.26	0.272
**CD163**	Stromal	0.7	0.72	0.032	0.08	0.26	0.344
	Tumoral	0.65	0.69	-0.042	0.17	0.36	0.132
	Whole core	0.32	0.39	-0.018	0.034	0.20	0.358
**FOXP3**	-	0.18	0.27	0.156	0.72	0.85	0.200
**Ki67**	Stromal	0.21	0.28	-0.005	0.89	0.92	0.152
	Tumoral	0.2	0.28	0.023	0.75	0.86	0.011
	Whole core	0.17	0.27	-0.028	0.71	0.85	-0.011
**TIM3**		0.47	0.54	0.038	0.46	0.61	0.180
**IDO1**		0.00018	0.00096	0.194	0.3	0.44	0.380
**ICOS**		0.000062	0.00077	0.141	0.013	0.16	0.327
**PD-L1 (SP142)**	Stromal	0.00092	0.0028	0.120	0.015	0.16	0.180
	Tumoral	0.0024	0.0059	0.092	0.24	0.38	0.336
	Whole core	0.0003	0.0014	0.123	0.11	0.27	0.195
**PD-L1 (SP263)**	Stromal	0.00081	0.0027	0.174	0.025	0.20	0.239
	Tumoral	0.0083	0.02	0.048	0.28	0.43	0.231
	Whole core	0.0017	0.0045	0.010	0.17	0.36	0.274

Significant p values are shown in red. The p-value were adjusted using the Benjamini Hochberg method.

Supplementary Table 6: Associations of predicted immune infiltrating populations with ER+ and ER- breast cancer

Cell type	ER+ p-value	ER+ adjusted p-value	ER- p-value	ER- adjusted p-value
**B cells**	0.023	0.05	0.79	0.98
**CD45**	0.016	0.04	0.34	0.84
**CD8**	0.012	0.04	0.12	0.84
**Cytotoxic T cell**	0.014	0.04	0.86	0.98
**Exhausted CD8**	0.16	0.23	0.98	0.98
**T cells**	0.00045	0.0036	0.84	0.98
**Th1 cells**	0.67	0.72	0.87	0.98
**Treg**	0.27	0.33	0.36	0.84
**DC**	0.038	0.07	0.95	0.98
**Macrophages**	0.05	0.08	0.35	0.84
**Mast cells**	0.26	0.33	0.4	0.84
**Neutrophils**	0.92	0.92	0.69	0.98
**NK CD56Dim**	0.44	0.50	0.36	0.84
**NK cell**	0.011	0.04	0.72	0.98
**M1/M2**	0.000031	0.00050	0.22	0.84
**CD68/CD8**	0.022	0.05	0.42	0.84

Significant p values on correction for multiple testing are shown in red for those associated with high pnSTING and in blue for those significantly associated with low pnSTING. The p-value were adjusted using the Benjamini Hochberg method

Supplementary Table 7: pnSTING^{low} gene signature

IMPA2
SLC2A4
CDT1
CFAP47
RABGGTB
NCAPD3
ABRA
SLC35C2
DNAH12
SLC35E2B
LINC00479
HYDIN2
CSN3
FAM71B
RCAN3
MSH2
PCDHA2
SHISA3
EXO1
GALNT14
VPS33A
DLGAP5
HFM1
BEND3
MEX3B

*Note: signature = -sum(gene expression)

Supplementary Table 8: pnSTING gene signature applied to independent datasets

**Gene Signature**		**N(n)**	**HR**	**%95 CI**	**p-value**
**Relapse Free Survival**			Univariate		
**ER+ IHC cohort**	low	46 (14)	1		
	high	46 (5)	0.3220	0.1309-0.7924	0.0217*
**ER- IHC cohort**	low	32 (9)	1		
	high	32 (8)	0.8985	0.3472-2.325	0.8254
**ER+ & ER- IHC cohort**	low	78 (24)	1		
	high	78 (12)	0.4402	0.2287-0.8471	0.0169*
**ER+ Non IHC cohort**	low	39 (10)	1		
	high	37 (3)	0.263	0.08845-0.7818	0.0263*
**ER- Non IHC cohort**	low	20 (9)	1		
	high	17 (8)	0.6018	0.2284-1.586	0.2896
**ER+ & ER- Non IHC cohort**	low	71 (18)	1		
	high	45 (12)	0.3126	0.1525-0.6409	0.0029**
**METABRIC (2012&2016)**		**N(n)**	**HR**	**%95 CI**	**p-value**
**Overall Survival**			Univariate		
**ER+**	low	486 (297)	1		
	high	485(262)	0.8098	0.6855-0.9650	0.0125*
**ER-**	low	167 (85)	1		
	high	166 (96)	1.258	0.9394-1.684	0.1201
**ER+ No chemo**	low	433 (270)	1		
	high	432 (240)	0.8322	0.6995-0.99	0.0379*
**ER+ Chemo**	low	53 (29)	1		
	high	53 (20)	0.5356	0.3046-0.9419	0.028*
**TCGA Nature 2012**		**N(n)**	**HR**	**%95 CI**	**p-value**
**Relapse Free Survival**			Univariate		
**ER+**	low	189 (25)	1		
	high	191 (20)	0.5481	0.3030-0.9953	0.0405*
**ER-**	low	59 (10)	1		
	high	59 (7)	1.441	0.5569-3.729	0.4251
**GSE2034 (Wang et al)**		**N(n)**	**HR**	**%95 CI**	**p-value**
**Relapse Free Survival**			Univariate		
**ER+**	low	104 (52)	1		
	high	105 (28)	0.4292	0.2761-0.6672	0.0002***
**ER-**	low	39 (10)	1		
	high	38 (17)	1.779	0.8364-3.783	0.1406

Supplementary Table 9: TP53, PIK3CA and MAP3K1 alterations in ER+ METABRIC and TCGA datasets stratified by pnSTING signature

Dataset	Gene	No. cases with alteration (%)		Log Ratio	p-value	q-value
**Mutations**		STING Sig low	STING Sig high			
**META-BRIC**	TP53	179 (36.83)	61 (12.58)	-1.55	<10 e-10	<10 e-10
	PIK3CA	185 (38.07)	268 (55.26)	0.54	5.26 e-8	4.549 e-6
	MAP3K1	31 (6.38)	83 (17.11)	1.42	1.15 e-7	6.61 e-6
**TCGA (Nature 2012)**	TP53	73 (38.02)	19 (9.95)	-1.93	5.09 e-11	4.40 e-7
	PIK3CA	61 (31.77)	94 (49.21)	0.63	3.596 e-4	0.577
	MAP3K1	11 (5.73)	27 (14.14)	1.3	4.55e-3	0.577

Supplementary Table 10: Copy number alterations of MYC and CCND1 in ER+ METABRIC and TCGA datasets stratified by pnSTING signature

Dataset	Gene	No Cases with alteration (%)		Log Ratio	p-value	q-value
**CNA**		STING Sig low	STING Sig high			
**META-BRIC**	MYC (8q24,21)	171 (35.19)	77 (15.88)	-1.15	<10 e-10	5.75 e-9
	CCND1 (11q13.3)	145 (29.63)	56 (11.55)	-1.37	<10 e-10	2.76 e-9
**TCGA (Nature 2012)**	MYC (8q24,21)	28 (14.81)	16 (8.42)	-0.81	0.0369	0.563
	CCND1 (11q13.3)	50 (26.46)	24 (12.63)	-1.07	5.035 e-4	0.166

Supplementary Table 11: Antibodies used in IHC

Antibody	***Clone***	***Company***	***Catalogue Number***	***Platform***	***Retrieval***	***Dilution***	***Incubation***	***Detection***
STING	Poly	Protein Tech	19851-1-AP	Leica Bond RX	ER1 20min<br>ER1 20min	1:1000<br>1:2000	15min @ RT<br>30min @ RT	DAB Polymer Refine + Enhancer<br>Opal Fluorophore 690
ER	6F11	Leica	NCL-L-ER-6F11	Leica Bond RX	ER2 30min	1:200	15min @ RT	DAB Polymer Refine + Enhancer
HER2	CB11	Leica	NCL-L-CB11	Leica Bond RX	ER1 25min	1:40	15min @ RT	DAB Polymer Refine + Enhancer
KI-67	30-9	Roche	790-4286	Ventana Benchmark XT	CC1 32min	Neat	16min @ 37C	Optiview DAB Kit
CK	AE1/AE3	Dako	M3515	Leica Bond RX	ER2 20min<br>ER2 20min	1:200<br>1:200	15min @RT<br>30min @RT	DAB Polymer Refine + Enhancer<br>Opal Fluorophore 520
CD3	2GV6	Roche	790-4341	Ventana Benchmark XT	CC1 32min	Neat	16min @ 37C	Optiview DAB Kit
CD4	SP35	Roche	790-4423	Ventana Benchmark XT	CC1 32min	Neat	16min @ 37C	Ultraview DAB Kit
CD8	C8/144B	Dako	M7103	Leica Bond RX	ER2 20min	1:200	15min @ RT	DAB Polymer Refine + Enhancer
CD20	L26	Dako	M0755	Leica Bond RX	ER1 30min	1:400	15min @ RT	DAB Polymer Refine + Enhancer
CD68	514H12	Leica	PA0273	Leica Bond RX	ER2 20min	1:200	30min @ RT	DAB Polymer Refine + Enhancer
CD45RO	UCHL1	Leica	NCL-L-UCHL1	Leica Bond RX	ER1 20min	1:2000	15min @ RT	DAB Polymer Refine + Enhancer
CD163	10D6	Leica	PA0090	Leica Bond RX	ER2 20min	Neat	15min @ RT	DAB Polymer Refine + Enhancer
FOXP3	SP97	LSBIO	LS-C210349	Leica Bond RX	ER2 20min	1:50	15min @ RT	DAB Polymer Refine + Enhancer
ICOS	D1K2T	Cell Signaling	#89601	Leica Bond RX	ER2 20min	1:400	15min @ RT	DAB Polymer Refine + Enhancer
IDO1	D5J4E	Cell Signaling	#86630	Leica Bond RX	ER2 20min	1:400	15min @ RT	DAB Polymer Refine + Enhancer
mTOR	49F9	Cell Signaling	#2976	Leica Bond RX	ER2 20min	1:100	15min @ RT	DAB Polymer Refine + Enhancer
PD-L1	SP263	Roche	790-4905	Ventana Benchmark XT	CC1 64min	Neat	16min @ 37C	Optiview DAB Kit
PD-L1	SP142	Spring Bioscience	M4420	Ventana Benchmark XT	CC1 64min	1:40	16min @ 37C	Optiview DAB Kit
TIM3	D5D5R	Cell Signaling	#45208	Leica Bond RX	ER2 20min	1:200	15min @ RT	DAB Polymer Refine + Enhancer
