# Supplementary material for: The clinical and molecular significance associated with STING signaling in breast cancer
Source: NPJ Breast Cancer. 2021 Jun 25;7:81. doi: 10.1038/s41523-021-00283-z (PMC8233333; doi:10.1038/s41523-021-00283-z)
Supplement: Supplementary file 2 — Reporting Summary [file 41523_2021_283_MOESM2_ESM.pdf]

nature research

Corresponding author(s): Niamh Buckley
Eileen Parkes

Last updated by author(s): Jan 7, 2021

Reporting Summary

Nature Research wishes to improve the reproducibility of the work that we publish. This form provides structure for consistency and transparency in reporting. For further information on Nature Research policies, see our Editorial Policies and the Editorial Policy Checklist.

Statistics

For all statistical analyses, confirm that the following items are present in the figure legend, table legend, main text, or Methods section.

n/a	Confirmed	
☐	☒	The exact sample size (n) for each experimental group/condition, given as a discrete number and unit of measurement
☐	☒	A statement on whether measurements were taken from distinct samples or whether the same sample was measured repeatedly
☐	☒	The statistical test(s) used AND whether they are one- or two-sided<br>*Only common tests should be described solely by name; describe more complex techniques in the Methods section.*
☐	☒	A description of all covariates tested
☐	☒	A description of any assumptions or corrections, such as tests of normality and adjustment for multiple comparisons
☐	☒	A full description of the statistical parameters including central tendency (e.g. means) or other basic estimates (e.g. regression coefficient) AND variation (e.g. standard deviation) or associated estimates of uncertainty (e.g. confidence intervals)
☐	☒	For null hypothesis testing, the test statistic (e.g. F, t, r) with confidence intervals, effect sizes, degrees of freedom and P value noted<br>*Give P values as exact values whenever suitable.*
☒	☐	For Bayesian analysis, information on the choice of priors and Markov chain Monte Carlo settings
☐	☒	For hierarchical and complex designs, identification of the appropriate level for tests and full reporting of outcomes
☐	☒	Estimates of effect sizes (e.g. Cohen's d, Pearson's r), indicating how they were calculated

Our web collection on statistics for biologists contains articles on many of the points above.

Software and code

Policy information about availability of computer code

Data collection	No software was used
Data analysis	No software was used

For manuscripts utilizing custom algorithms or software that are central to the research but not yet described in published literature, software must be made available to editors and reviewers. We strongly encourage code deposition in a community repository (e.g. GitHub). See the Nature Research guidelines for submitting code & software for further information.

Data

Policy information about availability of data

All manuscripts must include a data availability statement. This statement should provide the following information, where applicable:
- Accession codes, unique identifiers, or web links for publicly available datasets
- A list of figures that have associated raw data
- A description of any restrictions on data availability

All publicly available datasets are identified as indicated. Data is either available publicly or via the authors.

Field-specific reporting

Please select the one below that is the best fit for your research. If you are not sure, read the appropriate sections before making your selection.

☒ Life sciences ☐ Behavioural & social sciences ☐ Ecological, evolutionary & environmental sciences

For a reference copy of the document with all sections, see nature.com/documents/nr-reporting-summary-flat.pdf

Life sciences study design

All studies must disclose on these points even when the disclosure is negative.

Sample size	Sample size for the discovery dataset was determined by the available data, with statistical analysis plan pre-planned. No pre-determined power calculations were performed. Large publicly available datasets were utilised for validation.
Data exclusions	No data were excluded from analyses.
Replication	Data was independently verified using independent publicly available datasets (as referenced in the manuscript) and reproducible.
Randomization	Allocation was not random - multivariate analysis was performed as reported.
Blinding	Authors were blinded to pnSTING status of samples for scoring of immune markers and analysis of gene expression data.

Reporting for specific materials, systems and methods

We require information from authors about some types of materials, experimental systems and methods used in many studies. Here, indicate whether each material, system or method listed is relevant to your study. If you are not sure if a list item applies to your research, read the appropriate section before selecting a response.

Materials & experimental systems

n/a	Involved in the study
☐	☒ Antibodies
☐	☒ Eukaryotic cell lines
☒	☐ Palaeontology and archaeology
☒	☐ Animals and other organisms
☒	☐ Human research participants
☐	☒ Clinical data
☒	☐ Dual use research of concern

Methods

n/a	Involved in the study
☒	☐ ChIP-seq
☒	☐ Flow cytometry
☒	☐ MRI-based neuroimaging

Antibodies

Antibodies used

STING - 19851-1-AP ProteinTech
ER - NCL-L-ER-6F11, Leica
HER2 - NCL-L-CB11, Leica
Ki-67 - 790-4286, Roche
CK - M3515, Dako
CD3 - 790-4341, Roche
CD4 - 790-4423, Roche
CD8 - M7103, Dako
CD20 - M0755, Dako
CD68, PA0273, Leica
CD45RO - NCL-L-UCLH1, Leica
CD163 - PA0090, Leica
FoxP3 - LS-C210349, LSBio
ICOS - 89601, Cell Signalling
mTOR - 2976, Cell Signalling
IDO1 - 86630, Cell Signaling
PL-L1 - 790-4905, Roche
PD-L1, M4420, Spring Bioscience
TIM3 - 45208, Cell Signaling

See Supplementary table 1 for full list of antibodies and IHC protocols used in this study.

Validation

Antibodies have been validated using control tissues (tonsil, spleen, whole face tumour sections) and optimised in this manner.

April 2020

Validation	Antibodies are either diagnostic grade or validated by the manufacturer, in addition to quality control steps performed in house under the CRUK Accelerator programme.

Eukaryotic cell lines

Policy information about cell lines

Cell line source(s)	MDA-MB-436 EV - originally obtained from ATCC with sh-empty vector described in Parkes et al, JNCI, 2017.
Authentication	Authenticated as BRCA mutant previously (Parkes et al, JNCI, 2017)
Mycoplasma contamination	All cell lines tested negative for mycoplasma contamination and are tested on a regular basis.
Commonly misidentified lines (See ICLAC register)	Not relevant

Clinical data

Policy information about clinical studies

All manuscripts should comply with the ICMJE guidelines for publication of clinical research and a completed CONSORT checklist must be included with all submissions.

Clinical trial registration	*Provide the trial registration number from ClinicalTrials.gov or an equivalent agency.*
Study protocol	*Note where the full trial protocol can be accessed OR if not available, explain why.*
Data collection	*Describe the settings and locales of data collection, noting the time periods of recruitment and data collection.*
Outcomes	*Describe how you pre-defined primary and secondary outcome measures and how you assessed these measures.*
